# 3D cellular morphometrics of ovule primordium development in *Zea mays* reveal differential division and growth dynamics specifying megaspore mother cell singleness

**DOI:** 10.3389/fpls.2023.1174171

**Published:** 2023-05-12

**Authors:** Inès Ouedraogo, Marc Lartaud, Célia Baroux, Gabriella Mosca, Luciana Delgado, Oliver Leblanc, Jean-Luc Verdeil, Geneviève Conéjéro, Daphné Autran

**Affiliations:** ^1^ DIADE, University of Montpellier, IRD, CIRAD, Montpellier, France; ^2^ AGAP, University of Montpellier, CIRAD, INRAE, Institut SupAgro, Montpellier, France; ^3^ Institute of Plant and Microbial Biology, Zürich-Basel Plant Science Center, University of Zürich, Zürich, Switzerland; ^4^ IICAR, CONICET, University of Rosario, Rosario, Argentina; ^5^ IPSIM, University of Montpellier, CNRS, INRAE, Institut SupAgro, Montpellier, France

**Keywords:** ovule primordium, 3D morphometrics, developmental atlas, morphogenesis, *Zea mays*, female germ cell, MMC specification

## Abstract

**Introduction:**

Differentiation of spore mother cells marks the somatic-to-reproductive transition in higher plants. Spore mother cells are critical for fitness because they differentiate into gametes, leading to fertilization and seed formation. The female spore mother cell is called the megaspore mother cell (MMC) and is specified in the ovule primordium. The number of MMCs varies by species and genetic background, but in most cases, only a single mature MMC enters meiosis to form the embryo sac. Multiple candidate MMC precursor cells have been identified in both rice and *Arabidopsis*, so variability in MMC number is likely due to conserved early morphogenetic events. In *Arabidopsis*, the restriction of a single MMC *per* ovule, or MMC singleness, is determined by ovule geometry. To look for potential conservation of MMC ontogeny and specification mechanisms, we undertook a morphogenetic description of ovule primordium growth at cellular resolution in the model crop maize.

**Methods:**

We generated a collection of 48 three-dimensional (3D) ovule primordium images for five developmental stages, annotated for 11 cell types. Quantitative analysis of ovule and cell morphological descriptors allowed the reconstruction of a plausible developmental trajectory of the MMC and its neighbors.

**Results:**

The MMC is specified within a niche of enlarged, homogenous L2 cells, forming a pool of candidate archesporial (MMC progenitor) cells. A prevalent periclinal division of the uppermost central archesporial cell formed the apical MMC and the underlying cell, a presumptive stack cell. The MMC stopped dividing and expanded, acquiring an anisotropic, trapezoidal shape. By contrast, periclinal divisions continued in L2 neighbor cells, resulting in a single central MMC.

**Discussion:**

We propose a model where anisotropic ovule growth in maize drives L2 divisions and MMC elongation, coupling ovule geometry with MMC fate.

## 1 Introduction

Germ cells are the founder cells in sexually reproducing organisms. In both plants and animals, germ cell precursors are formed within a niche of somatic cells. In mammals, primordial germ cells are set aside in the embryo, and their specification is induced by signals from surrounding maternal tissues (Marlow, 2015). In flowering plants, spore mother cells differentiate in specialized floral tissues of adult plants (reviewed by Schmidt et al., 2015).

The plant female spore mother cell is called the megaspore mother cell (MMC). As early as the 19th century, differentiation of the MMC was recognized to occur specifically at the apex of ovule primordia (Hofmeister, 1849). In both dicot and monocot species, the MMC is characterized by its conserved position and morphology. The MMC occupies a central hypodermal position, has a large nucleus, and is enlarged and elongated compared to neighboring ovule cells (Russell, 1979; Huang & Sheridan, 1994; Schneitz et al., 1995; Lopez-Dee et al., 1999; Bhojwani & Soh, 2001; Lora et al., 2017; Mendocilla-Sato et al., 2017a; Hernandez-Lagana et al., 2021; Vijayan et al., 2021). The MMC is classically considered to derive from a progenitor cell, the archesporial cell. However, it is still controversial if this developmental transition involves species-specific or conserved processes. In some species, the MMC derives from a division of the archesporial cell (Bhojwani & Soh, 2001; Lora et al., 2017). In other species, like in *Zea mays*, conflicting studies report either a division of the archesporial cell or a direct differentiation of the MMC from the archesporial cell without division (Weatherwax, 1919; Cooper, 1937; Kiesselbach, 1949; Davis, 1966; Bedinger & Russel, 1994). The latter model was proposed in *Arabidopsis thaliana*, where no archesporial cell division was reported (Schmidt et al., 2015; Böwer and Schnittger, 2021).

Cell fate plasticity in the ovule nucellus is supported by genetic and molecular data showing that multiple MMCs or MMC-like cells can be formed in the ovule. For example, a pool of candidate archesporial cells have been identified in both *Arabidopsis* (Hernandez-Lagana et al., 2021) and rice (Nonomura et al., 2007), and some angiosperm species develop more than one MMC (Walters, 1962; Bachelier & Friedman, 2011; Lora et al., 2019). In *Arabidopsis*, natural variation exists for the number of MMC-like cells in ovule primordia, yet only a single embryo sac is formed (Rodríguez-Leal et al., 2015). This process involves one or several of the pathways known to restrict MMC fate to a single cell *per* ovule (MMC singleness) (Schmidt et al., 2015; Lora et al., 2019; Pinto et al., 2019; Böwer and Schnittger, 2021, Huang et al., 2022). Some of these pathways are remarkably conserved between *Arabidopsis* and grasses; for example, the RNA-directed DNA methylation (RdDM) pathway acts to control MMC singleness in both *Arabidopsis* and maize (Garcia-Aguilar et al., 2010; Petrella et al., 2021; Jiang & Zheng, 2022). Similarly, a signaling module involving the receptor *EXTRA SPOROGENOUS CELLS 1/EXCESS MICROSPOROCYTES 1* (*EXS1/EMS1*) and its ligand *TAPETUM DETERMINANT 1* (*TPD1*) restricts MMC number in maize and rice and pollen mother cell number in *Arabidopsis* (Sheridan et al., 1996; Sheridan et al., 1999; Zhao et al., 2002; Nonomura et al., 2003; Yang et al., 2003; Zhao et al., 2008; Wang et al., 2012). Also, mutations in ERECTA-like receptors generate multiple MMCs in both *Arabidopsis* (Hou et al., 2021) and rice (Zhao et al., 2020). In addition to these non-cell-autonomous control mechanisms, MMC fate plasticity is also controlled cell-autonomously *via* cell cycle regulation (Zhao et al., 2017). The current model involves thus both non-cell-autonomous and cell-autonomous control of MMC.

By contrast, our knowledge about the molecular control of MMC specification is scarce in both *Arabidopsis* and grass models. In *Arabidopsis*, MMC differentiation is promoted by *SPOROCYTELESS/NOZZLE* (*SPL/NZZ*) (Yang et al., 1999) and the downstream *WUSCHEL* (*WUS*) transcription factor (Lieber et al., 2011). This process involves a signaling module mediated by WINDHOSE1 (WIH1), WIH2, and the tetraspanin TORNADO2 (TRN2) (Lieber et al., 2011). In grasses, no specific MMC fate-promoting factor has been isolated. Instead, mutant screens revealed a handful of genes, including the NZZ/SPL homolog in rice, controlling the meiotic progression of the MMC but not its initiation (Golubovskaya et al., 1993; Nonomura et al., 2007; Pawlowski et al., 2009; Nonomura et al., 2011; Ren et al., 2018; Zhao et al., 2018).

In addition to these molecular controls, the geometry of the ovule has been shown to contribute to the regulation of MMC singleness in *Arabidopsis* by regulating the size of a competent domain in the subepidermal layer (L2) (Hernandez-Lagana et al., 2021). The role of anisotropic ovule growth was suggested by 3D tissue morphometric analysis, predicted by 2D growth models, and confirmed by quantitative analyses of *katanin* mutants affected in anisotropic cell growth: *katanin* mutant ovules showed altered ovule geometry with a notably enlarged dome, forming multiple MMC candidates (Hernandez-Lagana et al., 2021). Other observations suggested the existence of morphogenetic feedback between the somatic cell niche in the ovule primordium and MMC fate. For instance, in *Arabidopsis spl/nzz* mutants, MMC differentiation failure is associated with a reduction of nucellus growth (Balasubramanian & Schneitz, 2000). In maize anthers, *TPD1/MAC1* negatively controls pollen mother cell number but promotes cell division in the surrounding layers (Kelliher & Walbot, 2012; Wang et al., 2012). The action of *TPD1/MAC1* on ovule tissue around the MMC has not yet been studied.

Whether morphogenetic patterns associated with MMC plasticity in *Arabidopsis* are conserved in grasses is unknown. Here, we explored ovule primordium development in the sexual grass model *Z. mays*. Using 3D multiphoton imaging, semi-automated segmentation, and manual cell annotation, we produced a collection of 48 three-dimensional digital ovule primordia at five different stages, with cellular resolution. The analysis of morphogenetic descriptors allowed the reconstruction of a plausible developmental trajectory in the L2 niche of MMC founder cells. Starting from multiple candidate archesporial cells, the uppermost apical, central archespore undergoes a periclinal division that forms the MMC and an underlying “stack cell”. The MMC stops dividing, gradually expands, and acquires an anisotropic shape, while periclinal divisions are maintained in the L2 neighbors. This differential cell division results in MMC singleness. Concomitant to anisotropic ovule growth, differential expansion of the apical *vs*. basal cell walls of the MMC results in a trapezoidal shape, suggesting an organ-level control of the MMC shape. This work constitutes the earliest 3D quantitative description of the primary morphological cellular events associated with MMC formation in the ovule primordium in *Z. mays* and paves the way to functional studies of the conserved principles of MMC patterning in flowering plants.

## 2 Results

### 2.1 Building a 3D cellular morphometric atlas of maize ovule primordium development

**Figure 1:**
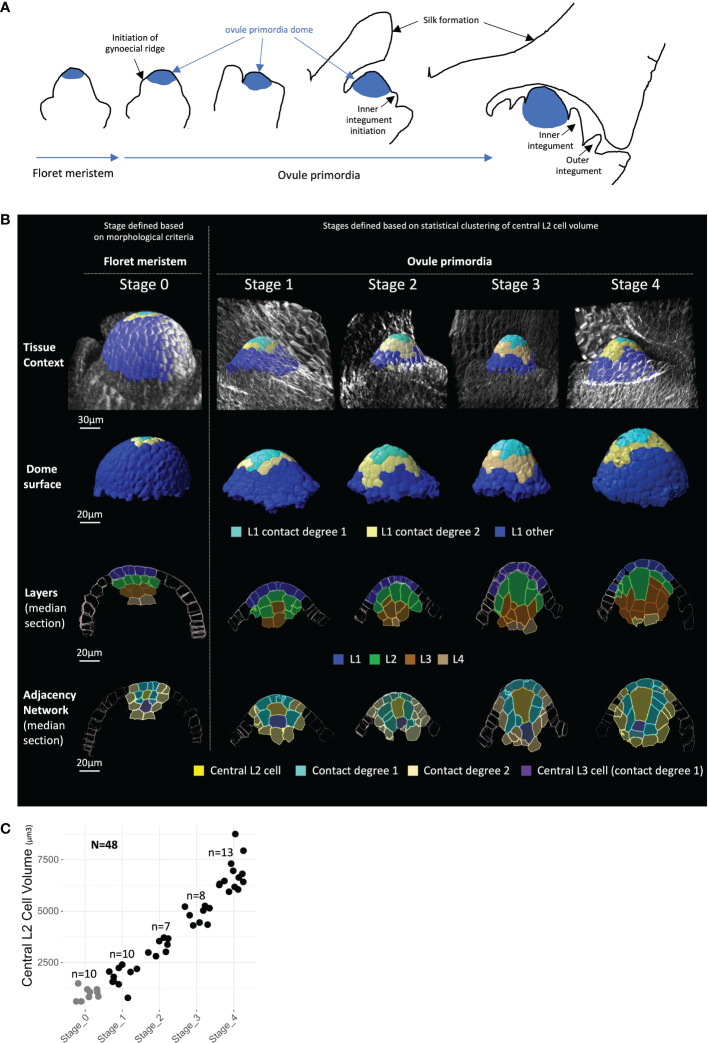
Building a 3D cellular morphometric atlas of maize ovule primordium development. **(A)** Schematic representation of maize ovule primordium formation from floret meristem and within the ovary context (drawn after Kiesselbach, 1949; Cheng et al., 1983; see also **Supplementary Figure 1**). **(B)** Representative 2D images of floret meristems (Stage 0) and ovule primordia (Stages 1 to 4) reconstructed in 3D, shown, respectively from top to bottom: in their tissue context; as isolated primordia dome; by longitudinal median sections labeled for cell layers, and labeled for radial position—i.e., the adjacency network of degree 2 centered on the most apical central L2 cell. Images were obtained from 3D reconstructions after cell segmentation was performed on multiphoton microscopy stacks of images. The color code for cell type labels is shown below image rows. A gallery of 2D images of full meristems and ovule primorda collection is presented in **Supplementary Dataset 1**, and full 3D image dataset is available in public repository (https://zenodo.org, *DOIs: 10.5281/zenodo.7671594*, *10.5281/zenodo.7675365*, and *10.5281/zenodo.7675409*). Scale bars: 30 μm for tissue context panel and 20 μm for other panels. **(C)** Ovule primordium Stages 1 to 4 defined by k-means clustering based on central L2 cell volume. Each dot represents central L2 cell volume for an individual ovule (black dots), or floret meristems (gray dots), while the latter were excluded from clustering analysis. N, total number of organs analyzed; n, number of meristems or ovule primordia analyzed in each stage.

To describe *Z. mays* ovule primordium development in 3D at a cellular level, we imaged a series of ovule primordia by multiphoton microscopy. Using 10 independent B73 plants, we generated a collection of fully segmented images in 3D (**Figures 1A, B**; **Supplementary Dataset 1**), capturing ovule primordia at different stages preceding the first meiotic division (**Supplementary Figure 1**). Fixed vibratome sections of young ears were cleared using ClearSee (Kurihara et al., 2015) and dual-stained using a cell wall dye and a DNA dye (see Materials and Methods). Image stacks capturing whole primordia (i.e., approximately 152 × 152 × (100–150) μm depending on organ size) were acquired with a cubic voxel resolution of 300 nm and used for 3D reconstruction and segmentation using IMARIS (Bitplane, Zürich, Switzerland). Semi-automatically segmented ovules were manually curated (Mendocilla-Sato and Baroux, 2017b and **Materials and Methods**). Thirty-eight images correspond to ovule primordia, identified by the formation of the surrounding gynoecial ridge, a structure that later forms the silk (**Figures 1A, B**; **Supplementary Figure 1**; see also Kiesselbach, 1949; Cheng et al., 1983; Irish, 1997).

For this image dataset, two overlapping levels of cell type annotation were generated (**Figure 1B**). Cell position within layers was annotated from the epidermis (L1 layer) to internal layers (up to L4), and cell types were further defined according to their radial position. We annotated the presumed MMC candidate as the most apical central L2 cell (called “*central L2 cell*”); adjacent cells were then annotated according to their degree of contact with the central L2 cell (“*contact 1*”, “*contact 2*”, and “*contact >2*”, **Figure 1B**). The L3 cell positioned below the central L2 cell was also annotated as a “*central L3 cell*”. This specific cell has been described in some grass species as the “stack cell”, the function of which is still unclear (Carman et al., 2011; Soliman et al., 2018; Colono et al., 2019). This annotation identified 11 distinct cell types for quantitative analysis of the ovule dome and the L2/L3 niche of cells involved in MMC formation. Among the 38 images, 25 were fully curated and annotated, while 13 images generated for analysis of the MMC and neighbor cells were annotated only for the L2 central cell and neighbor cells of contact degree 1. Finally, cellular descriptors of size and shape were exported for quantitative analysis of all annotated cells.

To objectively classify ovule primordia along a developmental trajectory for MMC growth, we used the volume of the central L2 cell as a criterion. Statistical clustering performed on this parameter allowed the identification of four groups, classified as Stages 1 to 4 and comprising 10, 7, 8, and 13 ovules, respectively (**Figure 1C**). The stages identified based on central L2 cell volume correctly reflected ovule growth, as assessed by additional growth criteria such as stage-by-stage increase of the mean cell number in L1 and L3 layers (**Supplementary Figure 2**). In addition, we generated, annotated, and quantified a set of 10 images corresponding to floret meristems preceding ovule primordium formation, hence called Stage 0. These meristems consist of a large dome of small cells, the uppermost of which will later contribute to the ovule primordium and where the gynoecium ridge is not yet visible (**Figures 1A, B**; **Supplementary Figure 1**; Kiesselbach, 1949; Cheng et al., 1983; Irish, 1997). Floret meristems (Stage 0) were excluded from clustering, as they were identified by independent morphological criteria. A gallery of all meristems and ovules is presented in **Supplementary Dataset 1**.

Overall, our dataset includes 7,369 cells from 48 ovules and floret meristems, reconstructed in 3D, annotated with positional information, quantified for size and shape descriptors, and classified into five developmental stages. This dataset constitutes the earliest comprehensive 3D description of ovule primordium in *Z. mays and* is available *via* a public repository (https://zenodo.org, *DOIs: 10.5281/zenodo.7671594*, *10.5281/zenodo.7675365*, and *10.5281/zenodo.7675409*). Here, we report an in-depth analysis of the primary morphological cellular events associated with MMC formation.

### 2.2 Maize MMC differentiates from an initial pool of enlarged hypodermal candidate archespores

**Figure 2:**
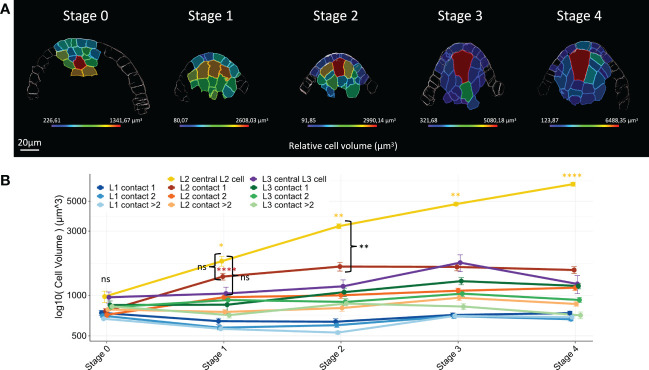
Maize MMC differentiates from an initial pool of enlarged hypodermal candidate archespores. **(A)** Representative heat maps of relative cell volume for floret meristems (Stage 0) and ovule primordia (Stages 1 to 4). The heat map color code (cell volume in μm^3^) is shown in the colored bar. Scale bar: 20 μm. Full heat map collection is presented in **Supplementary Dataset 1**. **(B)** Line chart plot comparisons of mean cell volume by cell type along developmental stages. Mean cell volume differences between groups were assessed using a Mann–Whitney U test. At Stage 0, no significant differences were detected between all cell types (ns). Yellow and red asterisks: significant size increase between two consecutive stages for the central L2 cell and L2 cells of contact 1. Significance is indicated above the latest stage. Black braces: comparison of central L2 cell with L2 cells of contact 1 and central L3 cell at Stages 1 and 2. No significant differences were detected at Stage 1 (ns), whereas central L2 cell volume increase is significantly different from Stage 2 onward. Error bars represent standard error (SE). Significance: p-value ≥0.05, ns; <0.05, *; <0.01, **; <0.001, ****. ns, not significant. Boxplot representation, including number of cells analyzed for each cell type, is presented in **Supplementary Figure 3**. Full statistical data are presented in **Supplementary Dataset 2**. MMC, megaspore mother cell.

In most angiosperms, the MMC occupies an apical position within the ovule dome, with increased cell size relative to all other ovule primordium cells (Hofmeister, 1849; Davis, 1966; Bhojwani & Soh, 2001; Lora et al., 2017; Hernandez-Lagana et al., 2021; Vijayan et al., 2021). To refine our analysis and identify when and how the maize MMC differentiates from the surrounding cells, we analyzed changes in cell volume of all annotated cells throughout primordium development. Consistently, we found that the MMC in maize shows the same striking morphological features as in other angiosperms: an apical position and increased size compared to surrounding cells (**Figure 2A**).

In meristems (Stage 0), all cell types have a comparable cell volume (**Figure 2B**; see also **Supplementary Figure 3** for boxplot representation and **Supplementary Dataset 2** for detailed p-values of statistical tests). Stage-by-stage comparisons showed that at Stage 1, L2 cells (except those of contact degree >2) displayed a significant size increase as compared to meristem (Stage 0) cells (**Figure 2B**). At later stages, cell size remained constant for all cell types except for the central L2 cell whose volume increased steadily, and for L1 cells at Stage 3. However, specific growth dynamics were observed for the L2 layer. As early as Stage 1, the central L2 cell and the L2 cells in contact degree 1 displayed a similar volume and were bigger than all L1 cells. However, from Stage 2 onward, while central L2 cells acquired a significantly higher volume than all other cell types, L2 cells in contact degree 1 retained a similar volume. This is possibly due to cell division that restores the initial cell size (**Figure 2A**). A similar growth dynamic was observed for central L3 cells. Finally, L2 cells in contact degree 2 were significantly smaller than their L2 neighbors in contact degree 1 up to Stage 3, although all L2 cells are generally bigger than L1 cells (**Figure 2A**; **Supplementary Dataset 2**).

Thus, cell volume analysis during ovule primordium development uncovered cell-specific growth trajectories according to position within the primordium. Our results show that the central L2 cell belongs to a group of cells with similar growth behavior from Stage 0 to Stage 1 (development of early ovule primordia), whereas from Stage 2 onward, the central L2 cell is distinguished by its increase in size. Collectively, the data suggest that the MMC differentiates from an initial group of morphologically homogenous cells, which may represent a pool of founder archesporial cells.

### 2.3 The maize MMC acquires an anisotropic trapezoidal shape during the late stages of ovule primordium development

We next asked whether the observed cell size dynamics were associated with specific cell shape patterns during ovule primordium growth. In particular, we aimed at determining i) if the presumptive MMC and its neighbor cells share similar cell shape dynamics at early stages (as they do for cell size) and ii) whether the MMC acquires a specific shape and if so at which stage.

**Figure 3:**
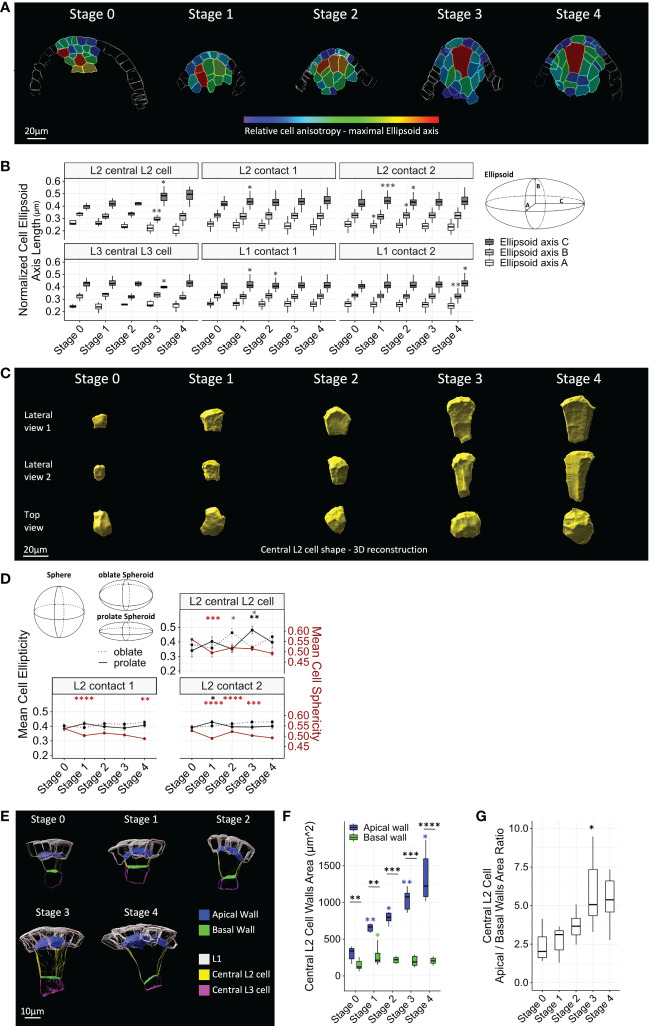
Maize MMC acquires an anisotropic trapezoidal shape at late stages of ovule primordium development. **(A)** Representative heat maps of relative cell anisotropy represented by the length of the major axis of the fitting ellipsoid for floret meristems (Stage 0) and ovule primordia (Stage 1 to Stage 4). The heat map color code is shown in the colored bar (major ellipsoid axis length in μm). Scale bar: 20 μm. Full heat map collection is presented in **Supplementary Dataset 1**. **(B)** Boxplot comparisons of mean normalized fitting ellipsoid axes length, by cell type, along developmental stages (left). Fitting ellipsoid axes, **(A–C)** are represented in the scheme (right). Asterisks: significant comparisons between two consecutive stages. **(C)** Representative 3D reconstructions of segmented central L2 cells throughout developmental stages, viewed from two perpendicular lateral sides (upper rows) and from above (top view, lower row). Scale bar: 20 μm. **(D)** Comparison of cell shape of L2 layer cell types using three shape cell descriptors, sphericity, prolate ellipticity, and oblate ellipticity, as represented in upper left corner. Color code for significance asterisks: red, cell sphericity; black, prolate ellipticity; gray, oblate ellipticity. Error bars represent SEM. **(E)** Representative images of segmented apical and basal walls of central L2 cells (surface of contact shared with L1 layer and central L3 cell, respectively) throughout developmental stages. Color codes are depicted in legend. Scale bar: 10 μm. **(F)** Boxplot comparisons of mean apical and basal wall areas of the central L2 cell throughout developmental stages. Asterisks above boxplots represent comparisons performed between two consecutive stages for each wall. Asterisks associated with brackets indicate comparison between apical and basal walls for each stage. **(G)** Comparison of ratios between apical wall area and basal wall area of the central L2 cell throughout developmental stages. For all graph panels: mean differences between two consecutive stages were assessed using a Mann–Whitney U test. Asterisks above boxplots represent comparisons performed between two consecutive stages. Significance is indicated above the latest stage. Significance: p-value ≥0.05, ns; <0.05, *; <0.01, **; <0.001, ***; <0.00, ****. Non-significant comparisons are not depicted to avoid overcrowding the figure. Full statistical data are presented in **Supplementary Dataset 2**. MMC, megaspore mother cell.

Cell shape changes were first assessed by characterizing the fitting ellipsoid of each cell. The normalized lengths of the fitting ellipsoid axes A (minimum), B (medium), and C (maximum) were calculated (**Figures 3A, B**). According to this descriptor, all ovule cells display some degree of anisotropic shape, since their maximum axis length was higher than either the minimum or medium axes lengths. However, when comparing the descriptor stage by stage, different dynamics in the acquisition of this anisotropic shape emerged. Most strikingly, the central L2 cell appeared as the most anisotropic cell in the ovule from Stage 3, with an ellipsoid axis C longer than that of the other cell types (**Figures 3A, B**; **Supplementary Dataset 2**). This increase in anisotropy at Stage 3 was characterized by a significant increase in axis C, with a simultaneous decrease in axis B, relative to Stage 2. These changes in shape were clearly visualized by 3D reconstructions of central L2 cells (**Figure 3C**). By contrast, L1 cells and L2 cells of contact degrees 1 and 2 displayed significant anisotropic growth changes up to Stage 2, yet no more significant change was observed from Stage 3 (**Figure 3B**). In addition, contrasting with other ovule cell types, anisotropy of the central L3 cell decreased significantly at Stage 3 (significant decrease of axis C coupled to a slight increase in axes A and B), i.e., simultaneous with the increase of anisotropy of the central L2 cell.

We observed a similar dynamic for three additional shape descriptors: sphericity and two ellipticity measures (i.e., oblate and prolate, corresponding to disk-shaped and oval or rugby ball-shaped spheroids, respectively). These descriptors were analyzed for the central L2 cell and its neighbors of contact degrees 1 and 2 (**Figure 3D**). Consistent with the anisotropy detected by the fitting ellipsoid descriptor, the characteristic elongation of the central L2 cell was visible at Stage 3 with a significant increase in prolate ellipticity and, consistently, a decrease in oblate ellipticity. Furthermore, a significant decrease in sphericity was observed at Stage 1 for all cells, which was maintained at later stages for central L2 cells and L2 cells of contact degree 1. In comparison, sphericity was more variable for L2 cells of contact degree 2.

To further describe the cell growth processes associated with the acquisition of a trapezoidal shape in the central L2 cell, we characterized the growth of the upper (apical) and lower (basal) cell walls. For this, we segmented the apical and basal cell wall areas and measured their respective size at different stages (**Figures 3E–G**). From the meristem stage onward, the apical wall was significantly larger than the basal wall. Moreover, while the basal wall expanded only between Stage 0 and Stage 1, the apical wall grew throughout development (**Figures 3E, F**). This differential expansion between the two walls was particularly marked between Stage 2 and Stage 3, as indicated by size ratio analysis (**Figure 3G**). To test if this trapezoidal shape *per se* is specific to the central L2 cell, at Stages 1 and 2, we measured the ratio of apical and basal wall areas of neighbor L2 cells of contact degree 1 (**Supplementary Figure 4**). We found a ratio not significantly different from the central L2 cell apical/basal wall ratio, yet L2 cells of contact degree 1 displayed a higher variability at Stage 1 (**Supplementary Figure 4**).

Overall, analysis of the dynamics of shape descriptors suggests that at early stages, the acquisition of anisotropic shape is not specific to the MMC, as neighboring cells are also anisotropically shaped. Thus, at early stages, directional growth appears coupled between the MMC and its neighbors. From Stage 3 onward, the MMC is the most anisotropic cell within the ovule, acquiring an oval/rugby-ball shape, indicating directional growth along a single main axis. This main axis is aligned with the axis of the ovule dome, as visualized in median longitudinal sections (**Figures 1A**, **2A**, **3A**; **Supplementary Dataset 1**). This axial growth of the MMC is concomitant with a significant expansion of its apical wall, while its basal wall stops growing, thereby generating a 3D trapezoid shape. Interestingly, the underlying L3 central cell maintains an isotropic shape as compared to other cell types in the ovule, and its growth arrest may contribute to limiting basal wall expansion of the MMC.

### 2.4 Differential pattern of periclinal divisions in L2 cells distinguishes the MMC from its neighbors

**Figure 4:**
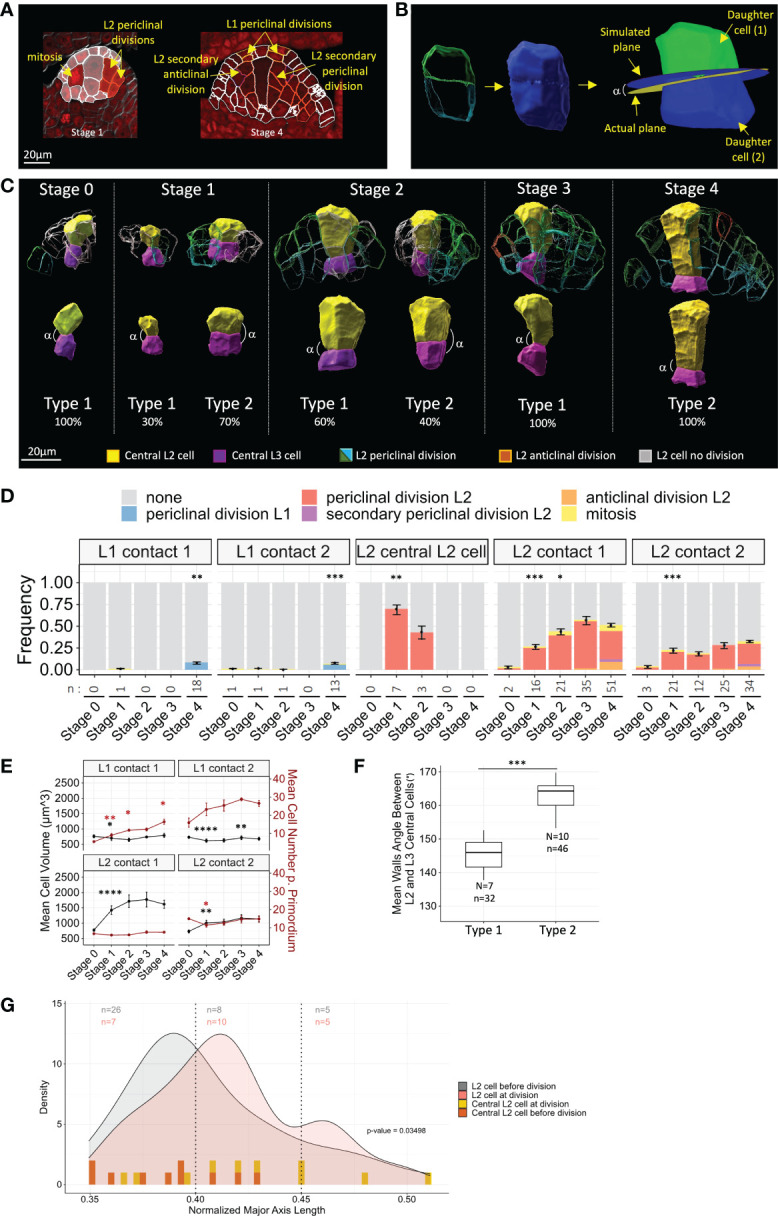
Differential timing of periclinal divisions in L2 cells distinguishes the MMC from its neighbors. **(A)** Examples of the different cell division types scored on representative 2D images. Segmented cell contours are outlined in red when cell division was observed or in white otherwise. Red nuclear signal: DNA dye propidium iodide. Scale bar: 20 μm. **(B)** Method for the verification of periclinal division prediction by 3D geometrical continuity using MorphoGraphX software. 3D meshes of observed cell pairs (left) were merged to obtain mother cell geometry (middle). Daughter cells were relabeled by cell tracking (right). Simulated geometrical cell division plane of mother cell was computed, together with its angle (α) with the actual cell division plane. **(C)** Representative 3D reconstructions of segmented central L2 (yellow) and L3 (magenta) cells displaying either geometrical continuity indicating recent division (Type 2 ovules) or disrupted geometrical continuity (Type 1 ovules), along developmental stages. Percentages indicate the proportion of Type 1 or 2 ovules observed at each stage. Examples of surrounding L2 cells, displaying divisions inferred from geometrical continuity, of periclinal orientation (green/blue contours, respectively, for L2/L3 daughter cells), secondary anticlinal divisions (red contours), or undivided cells (gray contours), are shown in upper panel. Extended gallery of Type 1 and Type 2 pairs of central L2/L3 cells is presented in **Supplementary Figure 6**. **(D)** Frequencies of cell divisions in L1 and L2 cells throughout developmental stages. Color codes for cell division types are depicted above chart. Statistical comparisons between consecutive stages were tested using a two-tailed Fischer’s exact test. Error bars represent SEM of cell numbers in non-dividing and dividing cell classes. n: number of cell divisions analyzed. **(E)** Mean cell volume (black lines and asterisks) and mean cell number per primordium (red lines and asterisks), in L1 and L2 cells of contact degrees 1 and 2. Mean differences between two consecutive stages were assessed using a Mann–Whitney U test (cell volume) and a two-tailed Fischer’s exact test (cell number). Error bars represent SEM. **(F)** Boxplot comparison of geometrical continuity of central L2 cell and central L3 cell quantified by mean wall angles between Type 1 and Type 2 ovules. N, number of central L2/L3 pairs analyzed. n, number of walls analyzed. Mean differences were assessed using a Mann–Whitney U test. **(G)** Comparison of cell anisotropy (major axis length) in L2 cells before division (gray curve) and at division (red curve) by density plots. For central L2 cells, anisotropy distribution before division (yellow bars) and at division (orange bars) is represented by bars for individual cells as density curves could not be drawn due to low sampling. Distribution heterogeneity was tested by χ^2^ test on three classes (p-value). n, number of cells analyzed for each class (color code following graph legend). For all graph panels: asterisks above bars or boxplots represent comparisons performed between two consecutive stages. Significance is indicated above the latest stage. Significance: p-value ≥0.05, ns; <0.05, *; <0.01, **; <0.001, ***; <0.001, ****. Non-significant comparisons are not depicted to avoid overcrowding the figure. Full statistical data are presented in **Supplementary Dataset 2**. MMC, megaspore mother cell.

We next explored whether specific cell division patterns are associated with MMC formation. To monitor the cell division rate, we first sought mitotic figures using the DNA signal (**Figure 4A**). However, these were too rare in our sampled dataset, probably due to the slow cell cycle and/or overall low division rate. To circumvent this issue, we rationalized that a geometrical continuity between the walls of two daughter cells should indicate a recent division event before the independent growth of the daughter cells creates deformations and loss of continuity (**Figures 4A–C**). Using this criterion, we were able to identify pairs of L2/L3 cells indicative of periclinal divisions at early stages in the L2 layer. At later stages, secondary cell divisions (i.e., occurring in previously divided cells) could be identified in either periclinal or anticlinal orientations. Periclinal divisions in L1 cells were also detected at Stage 3 and Stage 4. Examples of the different divisions identified are presented in **Figure 4A**.

To validate our approach, we verified that the identified pairs of cells corresponded to geometrically plausible cell division planes (**Figure 4B**). For this, we reconstructed *in silico* the predicted mother cell of pairs of daughter cells, using their 3D meshes. Then, using tracking and cell division plane computing functions of the MorphoGraphX software (Strauss et al., 2022), we computed the angle between the observed cell division plane and the predicted division plane by applying the geometrical rule of the shortest wall through the center of the cell, as described for symmetric divisions (Besson & Dumais, 2011) (**Figure 4B**). This angle (observed-predicted angle or o-p angle) was measured on a sample of 30 pairs of L2/L3 cells of contact degrees 1 and 2, distributed among stages and individual ovules. We found a mean o-p angle of 13.37° ( ± 1.33), indicating low divergence between observed and geometrically predicted division planes. For comparison, pairs of cells showing no 3D geometrical continuity showed o-p angles ranging from 9.27° to 41.33° (n = 5). Angles higher than 40° were reported only in mutant cells defective in cell plate orientation, such as *Tangled* in maize and *katanin* in *Arabidopsis* (Cleary & Smith, 1998; Webb et al., 2002; Uyttewaal et al., 2012). This approach confirmed that the criterion of cell wall continuity is a reliable indicator of recent cell divisions.

To obtain an estimation of the proliferation patterns in the ovule primordium, we then quantified periclinal division events, mitoses, and secondary periclinal and anticlinal division events (**Figure 4D**). We first asked whether divisions of the L2 cells surrounding the MMC could explain their previously observed constant volume (see **Figure 2**). Notably, we found that the division rate of L2 cells of contact degree 1 increased gradually during development and more significantly during the transition from Stage 1 to 2 (**Figure 4D**). This is consistent with the gradual increase of the summed number of L2 and L3 cells in contact degree 1 (**Supplementary Figure 5**), yet this measure is approximate since our labeling does not allow discriminating L3 cells arising from L2 divisions or L3 basal divisions. Divisions also occurred constantly in L2 cells of contact degree 2, from Stage 1 onward (**Figure 4D**). As expected, periclinal and anticlinal secondary divisions were restricted to late Stage 3 and Stage 4 ovules. At these late stages, periclinal divisions were also observed in the L1 layer (**Figure 4D**). However, the L1 cell number increased linearly from the early stages (**Figure 4E**). From these two observations, it can be inferred that L1 proliferation occurs mainly by anticlinal divisions at early stages. Following the same rationale, periclinal divisions of L2 cells reflect most of L2 proliferative activity, since L2 cell number did not increase significantly during development (**Figure 4E**).

We next asked whether periclinal division could also occur at early stages in the central L2 cell. Interestingly, 3D reconstructions revealed geometrical continuity between central L2 and central L3 cells in 70% and 42% of Stage 1 and Stage 2 ovules, respectively (**Figures 4C, D** and **Supplementary Figure 6**). Thus, periclinal division defines two types of ovules: Type 1 ovules showing no geometrical continuity between central L2 and central L3 cells and Type 2 ovules displaying geometrical continuity. We wished to confirm geometrical continuity in Type 2 ovules, but asymmetry in volumes of the central L2 and central L3 cells in some ovules made it impossible to observe and predict division planes (see below). Instead, we measured the angles between daughter cell walls on 3D reconstructions, reasoning that perfect continuity would lead to 180° angles between central L2 and central L3 cell walls (**Figure 4C**). Among Stage 1 and Stage 2 ovules, the mean angle in Type 2 ovules was closer to 180° and significantly higher than in Type 1 ovules (**Figure 4F**). The percentages of Type 2 ovules suggest that periclinal division in central L2 cells occurs preferentially at Stage 1 compared to Stage 2. There may be a certain level of plasticity in the timing of division for the central L2 cell, which showed however no further division from Stage 3 onward. Of note, though the anticlinal division of the central L2 cell was never observed, a single ovule at Stage 1 showed an oblique division plane (ovule 285, **Supplementary Dataset 1**). These results question the nature of the division of the central L2 cell: is it asymmetric or symmetric? In Type 2 ovules, we calculated the volume ratio between central L2 and central L3 cells and tested the heterogeneity using k-means clustering. We found one cluster of six ovules with a mean volume ratio close to 1 (cluster mean of 1.503), indicating a symmetric division. A second cluster comprised four ovules where the volumes of MMC and central L3 cells were different (ratio higher than 2, cluster mean of 2.341). This difference could be due either to an asymmetric division or to the differential growth of the two daughter cells following a symmetric division. Interestingly, Type 2 ovules at Stage 2 were all included in the second cluster, suggesting that if an asymmetric division occurred, this could have been favored by a larger size of the central L2 cell. However, the low sample size precludes a definitive analysis of the symmetry of the division of the central cell.

Finally, we asked whether cell division and cell shape are coupled in the ovule primordium. Can L2 periclinal divisions be driven by anisotropic growth? We first compared the distribution of shapes for L2 cells of contact degree 1 or 2, before and after a recent division (**Figure 4G**). The density distributions were significantly different (p-value = 0.03498 < 0.05; κ^2^ test) and showed a bias toward higher anisotropy for cells with recent divisions (**Figure 4G**). To test whether this coupling held true for the central L2 cell periclinal division, we compared the anisotropy of pairs of central L2 and central L3 cells from Type 2 ovules (n = 10), and of Stage 0 central L2 cells (n = 9), which had not yet divided. While shape variation was widely distributed for central L2 cells at division, the distribution of central L2 cells before division did not show such extreme anisotropy values and was concentrated in the first half of the value range (**Figure 4G**). Although further confirmation is needed because of the low number of ovules used, this analysis suggests that central L2 cells with high anisotropy are more likely to divide.

Taken together, our results suggest that divisions in maize ovule primordium are patterned in space and time. At early stages, ovule primordium cells divide mainly anticlinally in the L1 and periclinal in the L2. In a majority of ovule primordia, the MMC arises from a periclinally division, thus sharing lineage with the underlying L3 cell. After this division, the MMC stops dividing and starts expanding. By contrast, L2 neighbor cells display sustained proliferative activity. Developmental plasticity in the timing of division initiation possibly occurs for both MMC precursor division and neighbor cell divisions. However, it is also possible that neighbor cells initially divide more slowly than the MMC precursor, since they showed a lower division frequency at Stage 1. Interestingly, the initiation of division in L2 cells might be associated with increased anisotropy.

### 2.5 MMC anisotropic shape acquisition correlates with ovule primordium shape

**Figure 5:**
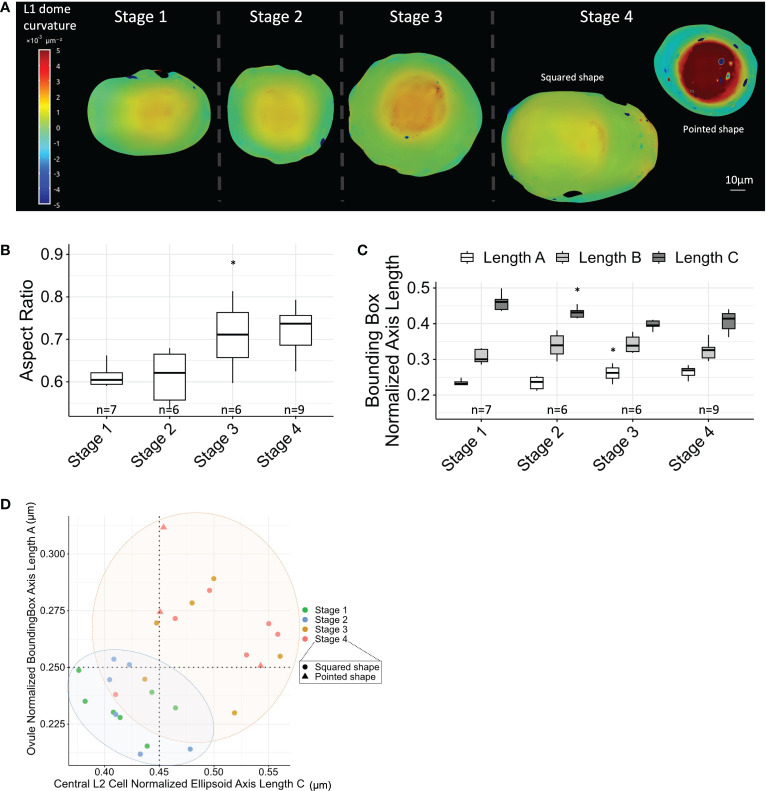
MMC anisotropic shape acquisition correlates with ovule primordium shape. **(A)** Ovule dome curvature analysis using L1 layer surface meshes, and computing curvature across the whole dome. Two types of ovules were observed at Stage 4: “pointed” ovules with high curvature and “squared” ovules with a curved ring surrounding ovule dome. Scale bar: 10 μm. Full collection of curvature maps is presented in **Supplementary Figure 7**. **(B)** Boxplot comparison of ovule aspect ratio (normalized ovule height extracted from fitting bounding box) throughout developmental stages. n, number of ovules analyzed. **(C)** Boxplot comparison of the normalized length of ovule axes (from fitting bounding box; length A representing ovule height, as verified from 3D visualization) throughout developmental stages. n, number of ovules analyzed. **(D)** Correlation between central L2 cell anisotropy (normalized fitting ellipsoid major axis length) and ovule anisotropy (aspect ratio) throughout developmental stages. Colored ellipses are visual help of two groups of stages, marking a shape transition between Stages 2 and 3. For all graph panels: mean differences between two consecutive stages were assessed using a Mann–Whitney U test. Asterisks above boxplots represent comparisons performed between two consecutive stages. Significance is indicated above the latest stage. Significance: p-value ≥0.05, ns; <0.05, *. Non-significant comparisons are not depicted to avoid overcrowding the figure. Full statistical data are presented in **Supplementary Dataset 2**. MMC, megaspore mother cell.

3D reconstructions of ovule domes suggested that ovule primordium shapes change throughout development (**Supplementary Dataset 1**). To better describe these changes, we analyzed the curvature of the ovule dome from Stages 1 to 4, using coarse surface meshes extracted from the L1 layer and computing curvature across the whole dome using MorphoGraphX. Curvature analysis was performed for a subset of ovules (see Materials and Methods). The analysis indicated increasing dome curvature with developmental progression (**Figure 5A**; **Supplementary Figure 7**). Moreover, at Stage 4, we observed two classes of ovule shape: i) “pointed” ovules with high dome curvature and ii) “squared” ovules with parallelepiped dome shapes (**Figure 5A**; **Supplementary Figure 7**, **Supplementary Dataset 1**).

To quantify the changes in the ovule dome shape, we computed a bounding box capturing the dome by its L1 layer and determined the normalized height/width ratio. This measurement indicated an increase in ovule dome height, simultaneous to width narrowing (**Figure 5B**). This conclusion was confirmed by the gradual increase of the minimal bounding box length A corresponding to ovule height (**Figure 5C**). To visualize whether the anisotropic growth of the MMC correlated with anisotropic ovule growth, we plotted the (normalized) maximum axis length of the central L2 cell against the ovule aspect ratio. This approach confirmed a global correlation of both MMC and ovule shape descriptors, with notably a shape transition between Stages 2 and 3 (**Figure 5D**).

In summary, maize ovule primordium growth is characterized by a gradual increase in anisotropy, partly explained by the formation of cell files through periclinal L2 cell divisions. Furthermore, oriented growth of the MMC and the ovule primordium appear coupled, with a major transition in shape between Stages 2 and 3.

## 3 Discussion

Plant organ morphogenesis results from coordinated cell growth and divisions. Previous work has shown that the shape of organs is relatively constant despite variability (plasticity) in cell size and shape between organs, a robustness attributed to adjustable feedback-based controls involving biochemical and mechanical signals (Bassel et al., 2014; Hong et al., 2018). However, how biochemical and mechanical signals are coordinated to define cell identities during organogenesis is still a current question in plant development (Landrein et al., 2015; Varapparambath et al., 2022). The plant ovule is an attractive morphogenetic model, where organ growth is coupled to the gradual acquisition of germline fate (Lora et al., 2017; Hernandez-Lagana et al., 2021; Vijayan et al., 2021). In the *Arabidopsis* ovule, growth achieves a specific, anisotropic, organ shape intimately linked to the differentiation of the MMC (Hernandez-Lagana et al., 2021).

Whether similar patterning rules govern MMC formation in grass models is not known. In maize, histological data on ovule development are mostly available for the late stages of MMC formation (Weatherwax, 1919; Cooper, 1937; Kiesselbach, 1949; Russell, 1979; Sheridan et al., 1996). Hence, we are missing a 3D understanding of ovule patterning at the early stages that are critical for MMC establishment. Here, we combine 3D imaging by multiphoton microscopy on cleared organs with image analysis to produce a repertoire of 3D digital maize ovule primordia, annotated at cellular resolution. We provide a detailed analysis of cellular descriptors that leads to a reference framework of ovule primordium patterning in this species.

Our results indicate that while the floret meristem is composed of homogenously sized cells, differential growth dynamics are established after ovule primordium initiation. At Stage 1, a pool of apical L2 cells enlarges and initiates periclinally cell divisions. In most ovules, the central L2 cell divides once periclinal at Stages 1 or 2 to generate the apical MMC and a basal daughter cell, the so-called “stack cell”. The MMC then stops dividing and starts expanding. Concomitantly, L2 neighbor cells show gradually increased division frequency, with proliferative activity maintained at late stages. Thus, differential division patterns between the L2 neighbor cells and the MMC result in the formation of a single MMC *per* ovule. In addition, periclinal divisions of L2 cells correlate with the establishment of anisotropic cell shapes. MMC trapezoidal shape was explained by the expansion of the apical cell wall following growth of the L1 (epidermis), while the basal cell wall did not significantly expand, constrained by the basal stack cell. This coupling suggests that organ-level control also determines MMC fate. This hypothesis is reinforced by the observation that anisotropic MMC shape acquisition correlates with anisotropic ovule growth along developmental stages. Below, we discuss several aspects of this sequence of cellular events, leading to the formation of a single MMC in the maize ovule primordium.

### 3.1 Conservation of a candidate pool of germ cells precursors in the maize ovule primordium

While the maize MMC was previously suggested to differentiate directly from a single precursor cell (Cooper, 1937; Kiesselbach, 1949; Davis, 1966; Bedinger & Russel, 1994; Sheridan et al., 1996; Golubovskaya et al., 1997), our analysis of early ovule stages suggested that the MMC actually differentiates from one of a pool of larger-sized apical L2 candidate precursors. This situation is consistent with recent studies of early ovule stages in *Arabidopsis* and rice (Nonomura et al., 2007; Hernandez-Lagana et al., 2021) and is reminiscent of animal primordial germ cells (Nguyen et al., 2019) and the pool of male meiocyte precursors in flowering plants (Marchant & Walbot, 2022). From the pool of germ cell precursors, a single MMC is formed in each ovule. Gradual canalization toward a single MMC was evidenced in *Arabidopsis* by 3D cell size and shape analyses (Hernandez-Lagana et al., 2021) and by molecular markers that mark a pool of precursors then specifically the MMC, such as the eviction of the histone HTR13 in *Arabidopsis* (Hernandez-Lagana & Autran, 2020) or MEL1 mRNA in rice (Nonomura et al., 2007; Yamaki et al., 2011). Furthermore, the identification of mutants able to form ectopic MMCs confirmed a shared competence among several L2 cells, which in the wild type is restricted to a single cell by several (conserved) pathways (Nonomura et al., 2003; Zhao et al., 2008; Nonomura, 2018; Pinto et al., 2019; Petrella et al., 2021; Huang et al., 2022; Jiang & Zheng, 2022). Maize mutants forming ectopic MMCs have also been identified (Sheridan et al., 1996; Garcia-Aguilar et al., 2010), consistent with our finding of a pool of candidate precursors. However, further investigations are needed to verify the gradual restriction of MMC identity to a single cell, using molecular markers such as maize *MEL1* homologs, *MAC1* (Kelliher & Walbot, 2012), or other pre-meiotic genes (Nelms & Walbot, 2019).

Collectively, these results suggest that the establishment of a pool of competent germline precursor cells on which gradual fate restriction is applied might be more prevalent in flowering plants than previously thought. It also raises the question of what mechanisms channel distinct developmental trajectories for the MMC and its L2 neighbors.

### 3.2 Does the maize MMC arise from a formative periclinal division?

We observed a periclinal division in the central L2 cell—the presumptive MMC—in most of the ovules we sampled. Does this event fit with the strict definition of an archesporial cell? Strictly defined, the term archesporial cell refers to the cell of the archesporium that undergoes a division forming a small cell and a larger cell that will become the spore mother cell (Davis, 1966; Schmidt et al., 2015; Böwer and Schnittger, 2021). This asymmetric division is formative since it generates two daughter cells with different fates (Petricka et al., 2009; Smolarkiewicz & Dhonukshe, 2013). The concept of archesporial cells applies to male PMC precursor cells since asymmetric formative divisions are observed in the anther lobes (Sanders et al., 1999; Marchant & Walbot, 2022). However, in the female germline, the use of this term remains largely uncertain, as the occurrence of a formative (and potentially asymmetric) division is not as clear as in pollen mother cell precursor cells (Böwer and Schnittger, 2021). In *Arabidopsis*, the MMC is usually described as differentiating directly from the (presumptive) archesporial cell. In contrast, in some species, periclinal divisions of the archesporial cell have been reported (Davis, 1966; Tilton and Lersten, 1980; Bhojwani & Soh, 2001; Lora et al., 2017). This feature is often linked with the crassinucellate nature of ovules—i.e., ovules developing multiple layers above the MMC, in contrast to a single layer in tenuinucellate ovules (Endress, 2011). Nevertheless, the formative nature of such division has not been demonstrated.

Maize ovules are classified as pseudo-crassinucellate—i.e., bearing a single epidermal layer at early stages, where periclinal divisions occur at later stages (Davis, 1966). Classical morphological descriptions have reported contradictory observations regarding archesporial cell division in maize ovules. Weatherwax (1919) described a periclinal division forming a transient apical parietal cell, which was not confirmed by Cooper (1937). Kiesselbach (1949) described both lateral and periclinal divisions of an enlarged subepidermal cell before it forms the archesporial cell and then direct differentiation of the MMC from the archesporial cell. Since then, the absence of division of the archesporial cell has been generally assumed in maize (Davis, 1966; Bedinger & Russel, 1994; Sheridan et al., 1996; Golubovskaya et al., 1997; Zhou et al., 2017). These discrepancies might come from the heterogeneity of the stages analyzed. Our study, which focusses on early stages, supports the occurrence of archesporial cell periclinal division in a majority of ovules in maize. Interestingly, the pattern of archesporial cell division observed here differs from the formation of the parietal cell, described previously in several species. The parietal cell is an apical, small, daughter cell, incorporated in L1, whereas the basal daughter cell forms the MMC (Weatherwax, 1919; Tilton, 1981; Raghavan, 1997). In this study, we observed that the apical daughter cell forms the MMC. Among species or genetic variants where archesporial cell division occurs, differential positional information mechanisms might explain whether the apical or basal daughter cell will form the MMC.

Here, we show that the division of the archesporial cell leads to two cells with different growth and shape, i.e., the expanding anisotropic MMC and its underlying isotropic small sister cell. It is thus reasonable to hypothesize that this is a formative division. However, whether this division is required for meiotic commitment remains to be functionally tested. Interestingly, a minority of ovules not showing evidence of archesporial cell periclinal division were also detected in our study. Although this can be due to low sample size, it might suggest that archesporial cell division is a plastic event, possibly depending on ovule tissue growth homeostasis signals feeding back to the archesporial cell. However, this hypothesis awaits further characterization by analyzing more ovules and using lineage tracing approaches.

### 3.3 The stack cell, formed by the division of the archesporial cell, contributes to the anisotropic growth of the MMC

Formative divisions have been proposed to provide daughter cells with different positional and polarity information (Smolarkiewicz & Dhonukshe, 2013). Here, we propose that a crucial outcome of the archesporial cell division is the formation of the central L3 cell beneath, the so-called “stack cell”, which has a key influence on MMC growth. Stack cells have been previously reported in grasses as enlarged cells at the basal pole of the MMC, generally proliferating at later stages into a file of cells, in both sexual species (Ren et al., 2018; Yang et al., 2022) and apomictic species (Leblanc et al., 1995; Carman et al., 2011; Soliman et al., 2018; Colono et al., 2019). However, stack cell formation was not studied at early developmental stages, nor was its potential lineage relationship with the archesporial cell.

Compared to the enlarged and anisotropic MMC, our results show that the stack cell is isotropic and restricted in growth. Strikingly, the MMC basal wall, shared with the stack cell, stops expanding rapidly. This suggests either material reinforcement of this wall or the absence of wall extensibility (Cosgrove, 2018; Aryal et al., 2020; Jonsson et al., 2022). In agreement with this, differential esterification of pectins has been recently shown in the basal cell wall of MMC in barley ovules, suggesting a higher wall stiffness than the apical one (Yang et al., 2022). This specific wall reinforcement might be crucial in generating the typical 3D trapezoidal shape of the MMC. In this perspective, the actual function of the archesporial cell (periclinal) division might be to provide, together with the stack cell, a positional cue in the organ, anchoring MMC expansion and possibly orienting ovule growth.

### 3.4 Coordination of anisotropic growth of the MMC and ovule

In addition to the growth restriction of its basal wall, the trapezoidal shape of the MMC is also achieved by the continuous expansion of its lateral walls (as inferred from its axial growth) and notably of its apical wall (as measured here). This suggests that MMC lateral and apical walls both have high extensibility. This might be achieved by low bundling of cellulose fibers (Cosgrove, 2018; Jonsson et al., 2022), enrichment in wall loosening proteins (see Lora et al., 2017 for examples in ovule cells; and Cosgrove, 2018; Jonsson et al., 2022 for examples in other plant cell types), or differential regulation of the hemicellulose matrix, as shown previously in ovules (Lora et al., 2017; Tucker et al., 2018; Yang et al., 2022). Thus, differential wall composition creating material anisotropy is predicted to control MMC anisotropic growth in the maize ovule.

Auxin, a major growth signal, is also likely involved in the control of MMC expansion and shape. Apical wall expansion of the MMC is strikingly coincident with the reported pattern of auxin response maxima (DR5 reporter) at the apex of the ovule, which is conserved in both *Arabidopsis* (Bencivenga et al., 2012; Yu et al., 2020) and maize (Lituiev et al., 2013). In addition, the auxin signaling reporter R2D2 demonstrated high auxin levels within the MMC in *Arabidopsis* (Kawamoto et al., 2020; Yu et al., 2020; Huang et al., 2022). Directional auxin flux is likely generated all along the epidermis and below the MMC in inner cells by PIN1 auxin transporter activity (Bencivenga et al., 2012), a pattern also conserved in maize (Lituiev et al., 2013). Interestingly, PIN1 polarity below the MMC might contribute to the growth restriction of the L3 stack cell by a yet-unknown mechanism.

Moreover, auxin is one of the mobile biochemical cues likely coordinating MMC and ovule growth. Other candidate coordinating signals include TPD1/MAC1 (Sheridan et al., 1996; Sheridan et al., 1999; Wang et al., 2012) and small RNAs (Komiya et al., 2014; Fei et al., 2016) shown to act in grasses to control germ cell fate. These biochemical cues act in concert with mechanical forces generated by the differential growth of the ovule cells connected through their walls of different material anisotropies. Such mechanical coordination is suggested by our results showing the striking dependency of MMC shape on surrounding cell growth dynamics, coupled with ovule shape acquisition. This coupling is consistent with 3D analyses in *Arabidopsis* wild-type and *katanin* ovule primordia, in which altered ovule shape changes MMC fate plasticity (Hernandez-Lagana et al., 2021).

However, the causal relationship between organ and cell shapes in the maize ovule is not known: does MMC growth follow the increase in height of the ovule primordium, or does the differential growth of the MMC drive the shape of the dome primordium? Quantitative analysis of ovule shapes in mutants displaying either multiple MMCs (Sheridan et al., 1996; Garcia-Aguilar et al., 2010) or reduced MMC growth (Golubovskaya et al., 1997) would help to distinguish between these alternatives.

### 3.5 Coordination of cell cycle control between the MMC and its neighbors

Our observations indicate that the timing of archesporial cell division is restricted to a short developmental window, while neighboring L2 cells maintain proliferation throughout the developmental stages analyzed. This differential control is consistent with previous cell cycle marker analyses in *Arabidopsis* showing that the MMC and its L2 neighbors are first in relative mitotic quiescence and then re-enter the mitotic cell cycle at later stages, while the MMC differentiates (Hernandez-Lagana & Autran, 2020; Hernandez-Lagana et al., 2021). This mechanism leads to a single central MMC. Furthermore, in *Arabidopsis* mutants developing ectopic MMC-like cells, divisions of L2 neighbors are delayed (Hernandez-Lagana et al., 2021). Thus, coordination of cell cycle control between the MMC and its neighbors’ likely controls MMC singleness. In maize, we observed that the timing of initiation of L2 neighbors’ divisions coincides with archesporial cell division but that L2 neighbors have a lower frequency of divisions at Stage 1. This indicates either a slower cell cycle or more variability from cell to cell or ovule to the ovule. This suggests either a division inhibitor mechanism acting at early stages in these cells or a division activating signal specifically in the central archesporial cell. The pool of founder L2 cells may initially have coupled regulation of growth and division, as shown in proliferative tissue such as the shoot apical meristem (Jones et al., 2017; D’Ario et al., 2021). In the MMC, the transition toward meiosis blocks the mitotic cycle and expansion starts, while in L2 neighbors, growth and division remain coupled. Interestingly, not only cell size but also cell shape seems to participate in the control of L2 cell division, since the more anisotropic cells appear, the more likely they are to divide. However, due to our low sampling for the central archesporial cell, this correlation needs confirmation.

### 3.6 Concluding remarks

Our morphometric analyses suggest conserved patterning principles leading to MMC formation between *Arabidopsis* and grasses. To verify the possible conservation of molecular processes, markers of cell fate, growth, and the cell cycle, as well as functional studies, are needed. Further perspectives to link cell size, shape, and cell fate in the ovule primordium include live imaging to monitor the dynamics of cell growth or high-throughput 3D analyses to describe the full cellular morphospace (Andrews et al., 2021).

Further deciphering the relationship between cell size, shape, and cell fate in grasses will be an important task to understand the transition between sexual and apomictic modes of reproduction (Schmidt, 2020). Indeed, some aposporic species are characterized by enlarged MMC neighbors (called aposporous initials) able to directly engage in gametogenesis. The resulting non-reduced egg cells form clonal seed progeny after parthenogenesis. By contrast, in diplosporous species, gametogenesis is triggered directly in MMCs and also leads to parthenogenetic, non-reduced egg cells. Interestingly, the growth of diplosporous reproductive cells is often significantly increased as compared to sexual spores, as shown in the apomictic maize relative *Tripsacum* (Leblanc et al., 1995). Therefore, altered growth of either the MMC or its neighbors is associated with diplosporic or aposporic modes of apomixis, respectively. Understanding how cell fate is controlled by ovule morphogenesis might be crucial to control apomixis, a process that offers important benefits for agriculture (Schmidt et al., 2015; Schmidt, 2020).

## 4 Materials and methods

### 4.1 Plant material

Seeds of the maize (*Z. mays*) B73 inbred line were germinated and grown to the seedling stage in a growth chamber and then transplanted in a greenhouse until ear emergence.

### 4.2 Imaging of maize ovule primordia

Young ears (ca. 0.8 to 1.5 cm) were embedded in 6% low melting point agarose and cut longitudinally using a vibratome (HM 650, Thermo Scientific, Walldorf, Germany) into 150- to 200-μm sections. Sections were transferred to multi-well plates and fixed overnight at 4°C in 4% paraformaldehyde + 0.1% Triton X-100 prepared in 10 mM of phosphate-buffered saline (PBS), rinsed twice in PBS, and cleared 10 days in the dark using ClearSee (Kurihara et al., 2015). DNA staining was performed overnight at room temperature with orbital stirring in the dark using 10 μg/ml of propidium iodide in ClearSee, followed by 5-h rinsing in ClearSee. Cell wall staining was performed (either before or after DNA staining) for 2 h at room temperature with orbital stirring in the dark using SR2200 Renaissance cellulose dye diluted 1,000× in ClearSee, followed by 1-h rinsing in ClearSee. Sections were mounted in ClearSee with spacers made with 170-μm-thick coverslips, covered with No. 1.5H coverslips.

Imaging was performed with an upright Zeiss 880 Multiphoton microscope (Zeiss, Jena, Germany) equipped with a pulsed laser Coherent Chameleon Ultra II (Coherent, CA, USA), a long-distance water dipping Plan-APO 20× 1.0 NA objective. Excitation was performed at 740 nm, and the emission signal was filtered using BP500-550 nm (Renaissance) and LP600 nm (propidium iodide) filters and collected directly on non-descanned NDD BIG detectors. The preview mosaic function was used to locate ovules along ear sections. Ovules at recorded positions were scanned (pixel dwell time of ca. 1 μs) in 3D, with settings adjusted to generate 0.3-μm cubic voxels stacks (image size of 150 × 150 μm in x, y, with a total stack thickness ranging from 100 to 130 μm, according to ovule stage), two times averaging, and automatic laser compensation to avoid signal decrease along the stack.

### 4.3 Image processing

Cell segmentation of 3D ovule primordia, and manual curation and corrections were performed in IMARIS (Bitplane, Switzerland) using procedures described in Mendocilla-Sato and Baroux, (2017b). Watershed segmentation was performed on the cell wall signal using automatic parameters, and the nuclear signal was used only for curation and corrections (ensuring that each segmented cell corresponds to a single nucleus). For segmentation corrections, the procedures described in Mendocilla-Sato and Baroux, (2017b) were used; alternatively, Cell objects were converted to Surface objects, and corrected using the “cut surface” tool, and then re-imported as Cell objects. Manual labeling of cell types was performed first for layers (from L1 to L4 layers; L4 cells were not quantitatively analyzed here). Then, radial positions were annotated, using 3D cutting planes, to identify first the most apical central L2 cell (label “*central L2 cell*”), and then adjacent cells were labeled at degree 1 and then 2 (labels: “*contact 1*”, “*contact 2*”, and “*contact >2*”). The L3 cell positioned below the central L2 cell was labeled as the “*central L3 cell*”. In a fraction of ovules, several L3 cells contacting the central L2 cell were detected; in such cases, the L3 cell best aligned with the axis of the central L2 cell was visually defined and labeled as the “*central L3 cell*”.

Representations by 3D renderings and heat maps were generated *via* combinations of IMARIS Surfaces (L1, central L2 or L3 cells, L2 cells with or without divisions) and Cell objects (adjacency network of degree 1 or 2, for heat maps), with or without cutting planes tool. Cell wall segmentation and contact area measurements were performed using the “Surface-Surface contact” Matlab Xtension in IMARIS. To generate whole ovule meshes for ovule shape quantification, L1 cells were selected, fused, and converted to a Surface object. Quantitative descriptors of either individual cells or whole ovules were exported as .csv files for further analysis.

For analyzing cell division plane orientation, 3D meshes were exported in .wrl format from Surface objects generated in IMARIS, converted to .obj format using Blender 3D rendering software (www.blender.org), and finally imported into MorphoGraphX (MGX) software (https://morphographx.org/software/). Pairs of 3D cells with predicted periclinal division were relabeled to identify mother and daughter cells; the simulated cell division plane was computed, and the angle between the actual and simulated plane was calculated, using the processes “Division analysis”, “Division Plane Approximation”, “Compute Division Plane Angles”, and “Display and Filter Planes”. To generate ovule dome curvature heat maps, ovule L1 cells 3D meshes were exported in .wrl format from Surface objects generated in IMARIS and imported in MGX as described above. Meshes without local irregularities were selected for further analysis. Curvature was computed and projected on simplified smoothed surface meshes using the MGX process “Project Mesh Curvature”, using a neighborhood distance of 40 µm; to avoid edge effects, this distance was reduced to 30 µm for small diameter ovules.

### 4.4 Quantitative analysis

Quantitative analysis of exported cell descriptors and cell numbers was performed essentially in R (R core Team, 2021; https://www.R-project.org/).

#### 4.4.1 Cell and organ descriptor measurements

Cell volume and ellipticity (oblate and prolate) were directly analyzed for the different cell types and stages. The IMARIS exported measures (object-oriented) “fitting ellipsoid axis lengths” A (minimum), B (medium), or C (maximum) were normalized by the sum of the three lengths, for cell anisotropy quantification. The ovule aspect ratio was calculated as A/(A+B+C) since A (minimum axis) always corresponded to the height of the ovule (B and C being the diameters of the ellipsoid shape of the base of the ovule). MGX was used to compare cell anisotropy between non-divided and divided cells: reconstructed mother cells of pairs of divided cells (see above) and meshes exported from IMARIS for non-divided cells were measured using the processes “Measures 3D/Geometry/Length Major Axis and/Length Minor Axis”, which compute minimal and maximal internal Eigenvalues (Barbier de Reuille et al., 2015). The major axis was then normalized by the sum of the two axes. Measurements of the walls’ angle in central L2/L3 pairs of cells were performed on 3D reconstructions, using the IMARIS tool “Measurement points”, by manually placing 3 points: one at the center of each wall of L2 and L3 cells and one at the junction between the two walls. This defined two segments, whose angle was calculated automatically by the software.

#### 4.4.2 Statistical analysis

For ovule stage classification, we used an unsupervised classification method, based on central L2 volume as a continuous variable (which increases linearly along development and is the focus of this study). The optimal number of clusters in the dataset was first determined using the Elbow method, giving the total Within Cluster Sums of Square (WSS) as a function of the number of clusters. This was performed using the function fviz_nbclust() and the parameter method=“wss” from the “factoextra” R package, taking central L2 cell volume as the classifier. Then, the partitioning clustering was performed using unsupervised k-means clustering, using the function kmeans() specifying the parameter k (the number of clusters) from the “stats” package, over the central L2 cell volume parameter.

For statistical comparisons, because of the low number of specific cell types at certain developmental stages (n < 10 for the central L2 and L3 cells), the normality of the data could not be assumed, and thus non-parametric tests were used. To determine if the means between two independent samples were significantly different, Mann–Whitney U two-tailed tests were performed using the wilcox_test() function from the “rstatix” package (p-value < 0.05). Two-tailed Fisher’s exact test (fisher_test() function of “rstatix”; p-value<0.05) was performed on the count of divided and non-divided cells to identify consecutive stages with different proliferation rates, or on cell number counts between stages. Graphical representations were performed using the “ggplot2” and “ggboxplot” R packages. For all statistics, the sample sizes, their means, and standard errors (SEM), as well as the p-values of the different statistical tests, are reported in **Supplementary Dataset 2**.

## Data availability statement

The datasets presented in this study can be found in online repositories. The names of the repository/repositories and accession number(s) can be found below: https://zenodo.org, DOIs: 10.5281/zenodo.7671594, 10.5281/zenodo.7675365, 10.5281/zenodo.7675409.

## Author contributions

IO: conceptualization, formal analysis, methodology, validation, visualization, writing—original draft preparation, and writing—review and editing. ML: investigation, methodology, and data curation. CB: conceptualization, methodology, supervision, resources, funding acquisition, and writing—review and editing. GM: conceptualization and methodology. LD: funding acquisition and writing—review and editing. OL: funding acquisition, project administration, and writing—review and editing. J-LV: funding acquisition, and resources. GC: investigation, methodology, data curation, funding acquisition, and writing—review and editing. DA: conceptualization, investigation, methodology, data curation, formal analysis, validation, visualization, supervision, funding acquisition, project administration, writing—original draft preparation, and writing—review and editing. All authors contributed to the article and approved the submitted version.

## Supplementary material

Supplementary Figures Supplementary figures 1 to 7 (pdf file).

Supplementary Dataset 1 Gallery of complete image collection of meristems and ovule primordia (pdf file).

Supplementary Dataset 2 full statistical data (Excell file). The dataset Image collection of 3D segmented Maize ovule primordia (B73 genotype)] for this study can be found in the public repository at (https://zenodo.org, *DOIs: 10.5281/zenodo.7671594, 10.5281/zenodo.7675365 and 10.5281/zenodo.7675409.*
