# Supplementary material for: 3D cellular morphometrics of ovule primordium development in Zea mays reveal differential division and growth dynamics specifying megaspore mother cell singleness
Source: Front Plant Sci. 2023 May 12;14:1174171. doi: 10.3389/fpls.2023.1174171 (PMC10213557; doi:10.3389/fpls.2023.1174171)
Supplement: Supplementary Figures — 1 to 7 (pdf file). [file DataSheet_1.zip › Supplementary Material/Ouedraogo_et_al_Supplementary_Figures.pdf]

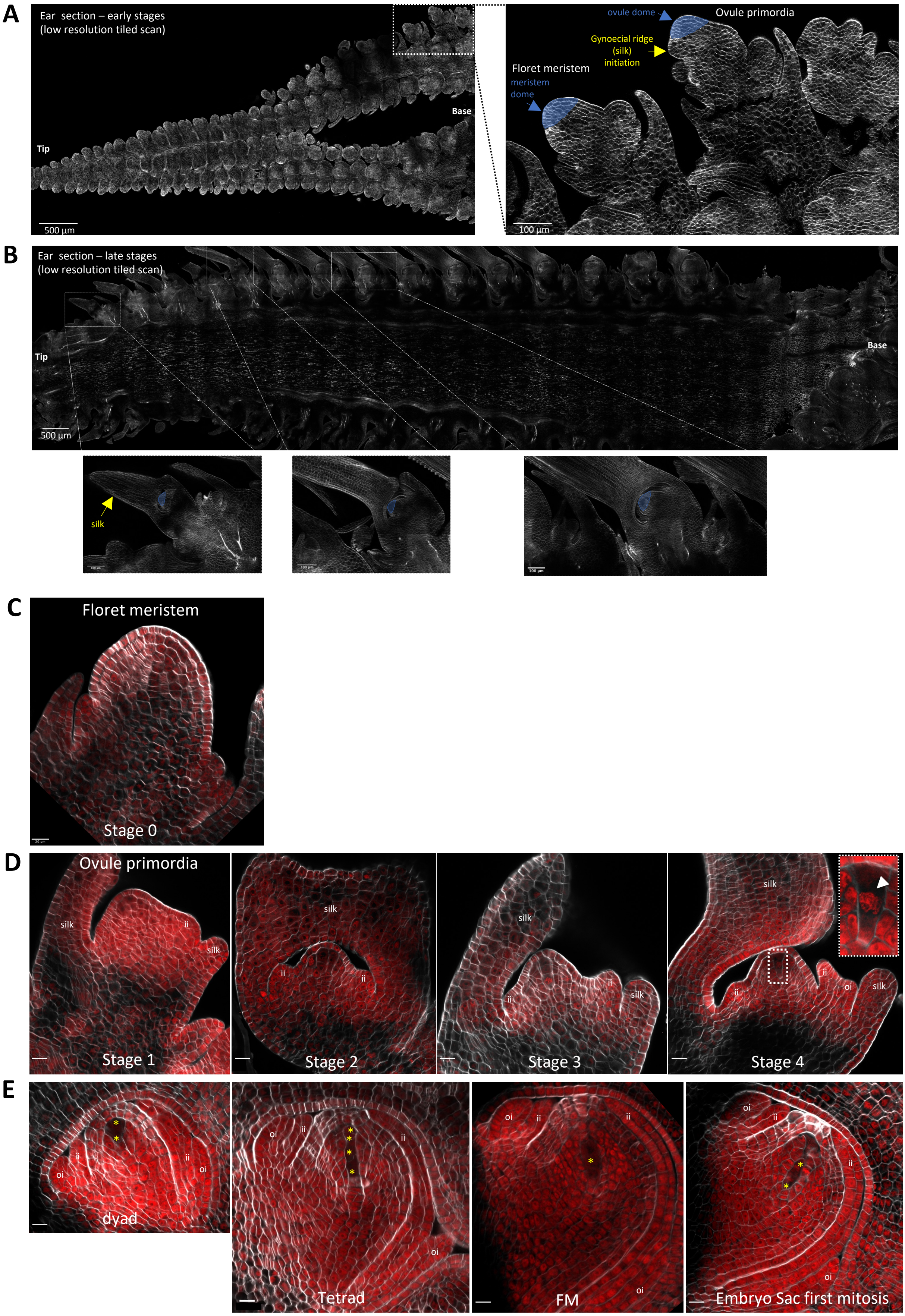

F

	Stage 1		Stage 2		Stage 3		Stage 4	
	Min.	Max.	Min.	Max.	Min.	Max.	Min.	Max.
**Central L2 cell Volume** (μm^3)	1567	2402	2810	3712	4345	5219	6178	8750
**Primordium Volume** (mm^3)	98,6	257,6	127,1	215,2	195,9	324,0	200,2	399,7
**Ear size** (mm)	9,2	13	9,2	14,9	9,8	13,2	9,8	14,9
**Trend ovule position along ear**	base	top	middle	top	base	middle	base	middle

Supplementary figure 1. Representative images of the developmental window covered by the study.

Supplementary figure 1. Representative images of developmental window covered by the study. (continued).

(A) Example of early stage ear section (stained for cell walls) and magnification of representative examples of consecutive floret meristem (absence of gynoecial ridge or silk initiation), and early ovule primordia (gynoecial ridge initiation is visible, yellow arrow). Organs' apical domes, where the archespores form, are highlighted in blue. Scale bars : 500 µm (left) and 100 µm (right), as indicated on pictures.

(B) Example of late stage ear section, and magnification of representative examples of consecutive ovule primordia showing a developmental gradient toward the base of the ear. Silk (yellow arrow), and ovule dome (blue highlight) are indicated. Scale bars: 500 µm (top) and 100 µm (bottom), as indicated on pictures.

(C) Example of floret meristem, before ovule silk initiation, defined here as Stage 0. High resolution image after staining of cell walls (white signal) and nuclei (red signal). Scale bar : 20 µm.

(D) Examples of ovule primordia of the four developmental stages (defined by central L2 cell volume statistical clustering) which were analyzed quantitatively in this study. At Stage 4, meiotic chromosomes figures (arrow in inset image) could be detected in some ovules, this group thus encompasses meiotic prophase I. Silk, inner integument (ii) and outer integument (io) are indicated. Scale bars : 20 µm.

(E) Examples of subsequent stages of ovule development not analyzed in this study, i.e. ovules after meiosis I (dyad) and II (tetrad), and at early gametogenesis, harboring a Functional Megaspore (FM), or two-nuclei embryo sac. Asterisks indicate individual nuclei in spores and gametophyte. Note multiple periclinal divisions in L1 and ovule teguments growth also characterizing these developmental stages. Inner integument (ii) and outer integument (io) are indicated. Scale bars : 20µm.

(F) Ranges (minimum and maximum values) of central L2 cell volume, primordium volume, ear size and position of ovules along the ear, from Stage 1 to Stage 4. Primordium volume was estimated by the volume of the fitting ellipsoid extracted from primordia domes meshes. Ear size was estimated from tile-scans ear section images as in (A). Ovule position along the ear are given as general trends, given the variability of the positions of the ovules actually analyzed and staged (not all ovules along a ear could be analyzed mainly due to initial vibratome section orientation, limiting complete ovule imaging).

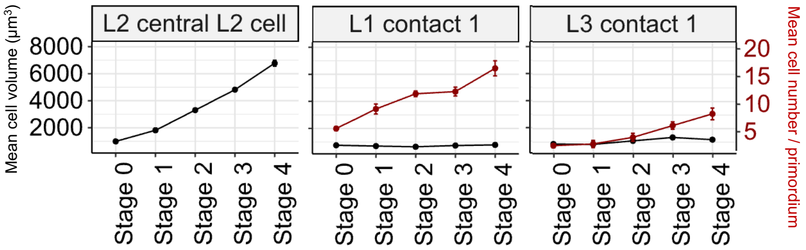

Supplementary figure 2. Positive ovule growth criteria along defined developmental stages.

Developmental stages were defined based on morphological criteria for Stage 0 florets meristems, and on central L2 cell volume statistical clustering for ovule primordia from Stage 1 to 4. Along these developmental stages; mean central L2 volume (left panel) increases linearly by definition. Cell number *per* primordium (red) in L1 cells of contact degree 1 (middle panel); and L3 cells of contact degree 1 (right panel) also showed a gradual increase along defined developmental stages, confirming positive ovule growth. Consistently, mean cell volume in L1 and L3 cells of contact degree 1 was maintained constant because of cell divisions. Error bars represent SEM.

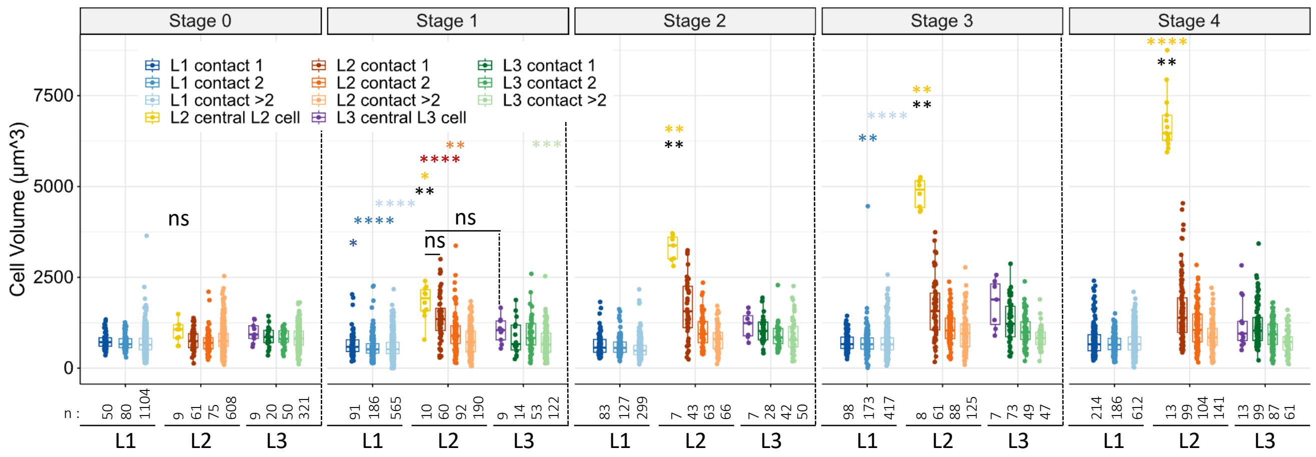

Supplementary figure 3. Boxplot representation of cell volume comparison by cell type along developmental stages.

Mean cell volume differences between groups were assessed using a Mann Whitney U test. Colored asterisks: significant comparisons between two consecutive stages of a same cell type, using cell type color code. Significance is indicated above the latest stage. Black asterisks: comparison between cell types for each stage: for this comparison, only statistical tests involving the central L2 cell are represented, to avoid overcrowding of the figure.

Significance: p-value ≥ 0.05: ns; < 0.05: *; < 0.01: ** ; < 0.001: *** ; < 0.001: ****. ns: non significant. n: number of cells analyzed for each cell type.

Full statistical data are presented in Supplementary dataset 2.

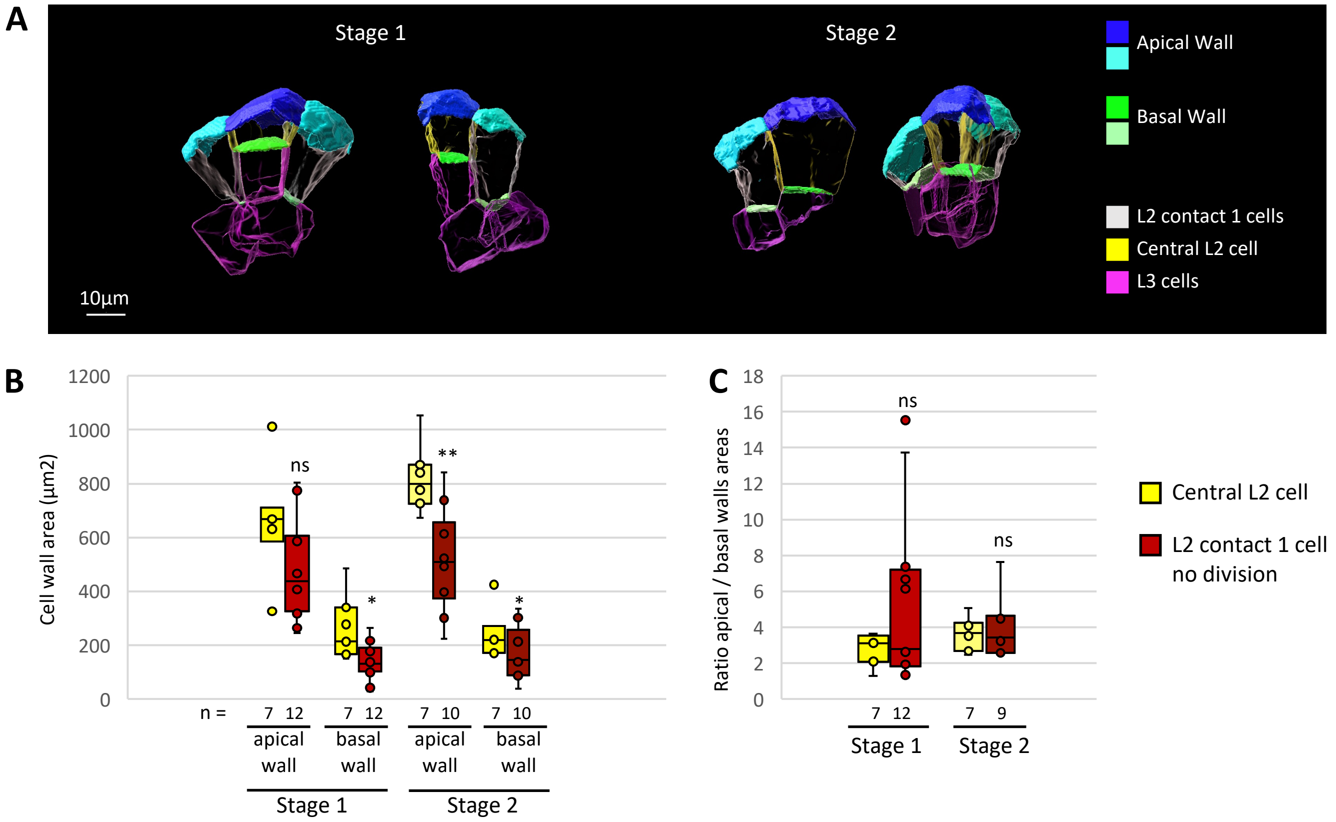

Supplementary figure 4. Comparison of apical and basal walls differential growth in the central L2 cell *versus* its L2 neighbors cells.

(A) Representative 3D reconstructions showing apical and basal walls surfaces of L2 cells of contact degree 1 (before division), of central L2 cell and underlying L3 cells; at Stages 1 and 2 (i.e. when non dividing L2 cells of contact degree 1 are still present). Color codes of walls and cell types as depicted in legend. Scale bar: 10µm.

(B) Boxplots comparison of apical and basal walls areas, in L2 cells of contact degree 1 (red) and central L2 cell (yellow).

(C) Boxplots comparison of apical / basal wall area ratios in L2 cells of contact degree 1 (red) and central L2 cell (yellow).

Mean differences were assessed using a Mann Whitney U test. Significance: P-value ≥ 0.05: ns; < 0.05: * ; < 0.01 : **. ns: non significant. n : number of cells/walls analyzed.

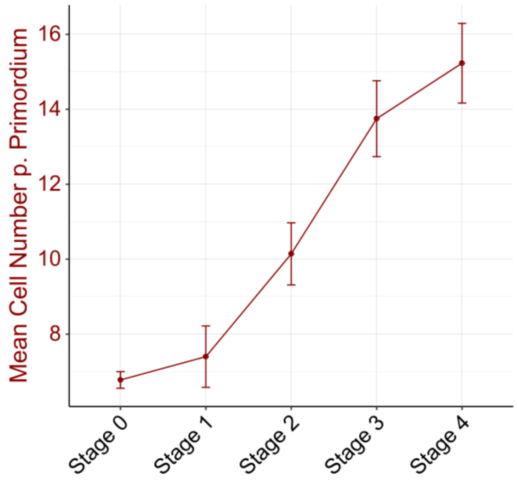

Supplementary figure 5. Mean cell number *per* primordium in L2 and L3 cells of contact degree 1. Cell number in L2 and L3 cells of contact 1 was calculated to estimate MMC's neighbors divisions. This parameter increases gradually during ovule primordium growth, and changes MMC topology. For Stage 0 florets meristems, only L2 cells of contact degree 1 were taken into account, given the absence of periclinal divisions in these cells.

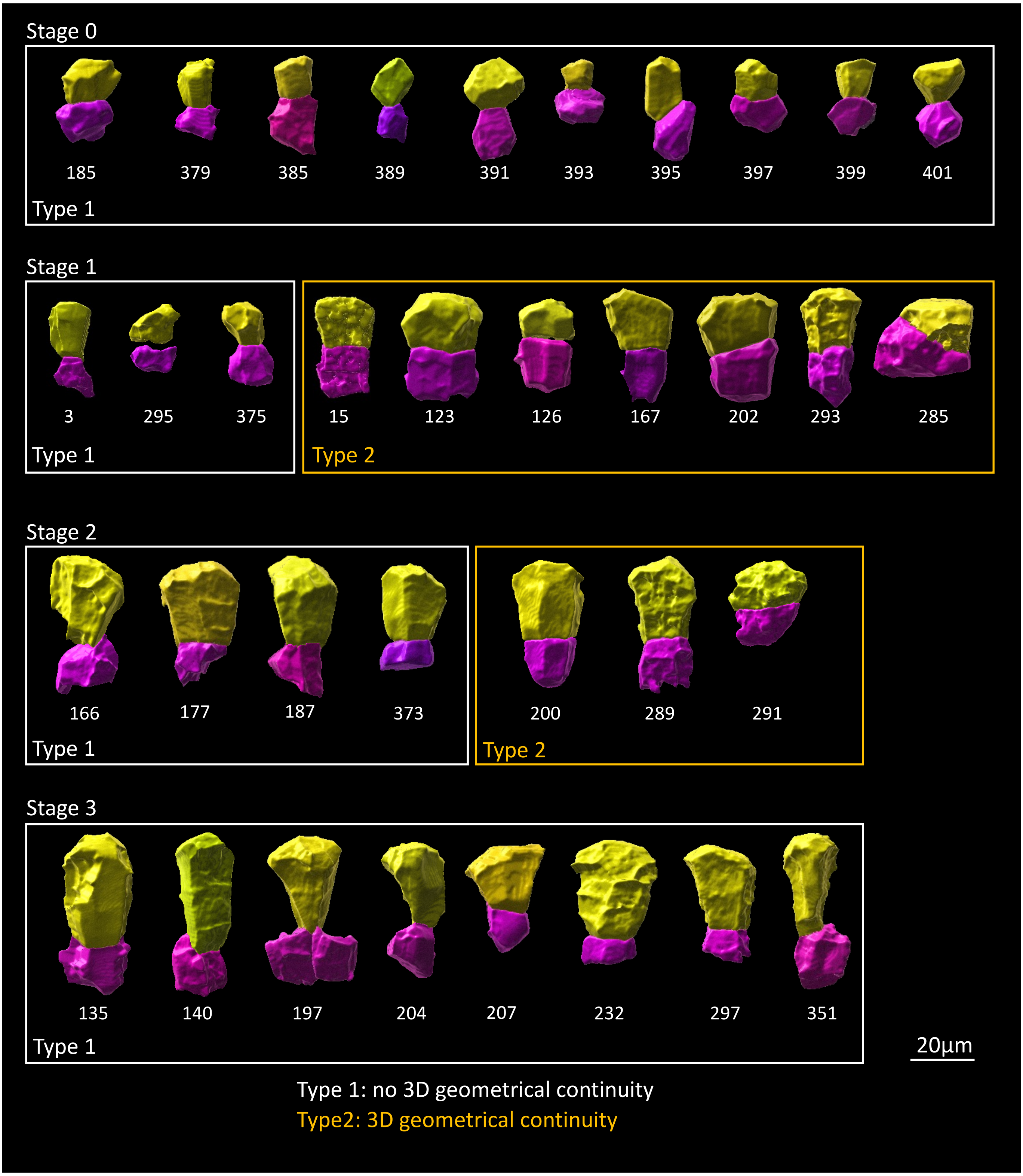

Supplementary figure 6. 3D reconstructions of central L2 and L3 cells pairs classified as Type 1 (geometrical continuity between cells is not observed) or Type 2 (geometrical continuity is observed, indicating recent periclinal division).

For each developmental stage, florets meristems or ovules displaying 3D geometrical continuity between central L2 cell (yellow) and underlying L3 cells (magenta) are depicted as Type 2 (orange squares), while Type 1 (white squares) organs display no observed geometrical continuity in 3D. Stage 4 ovules were all classified as Type 1 (not shown). Numbers below images indicate organs' image stack ID. Scale bar: 20µm.

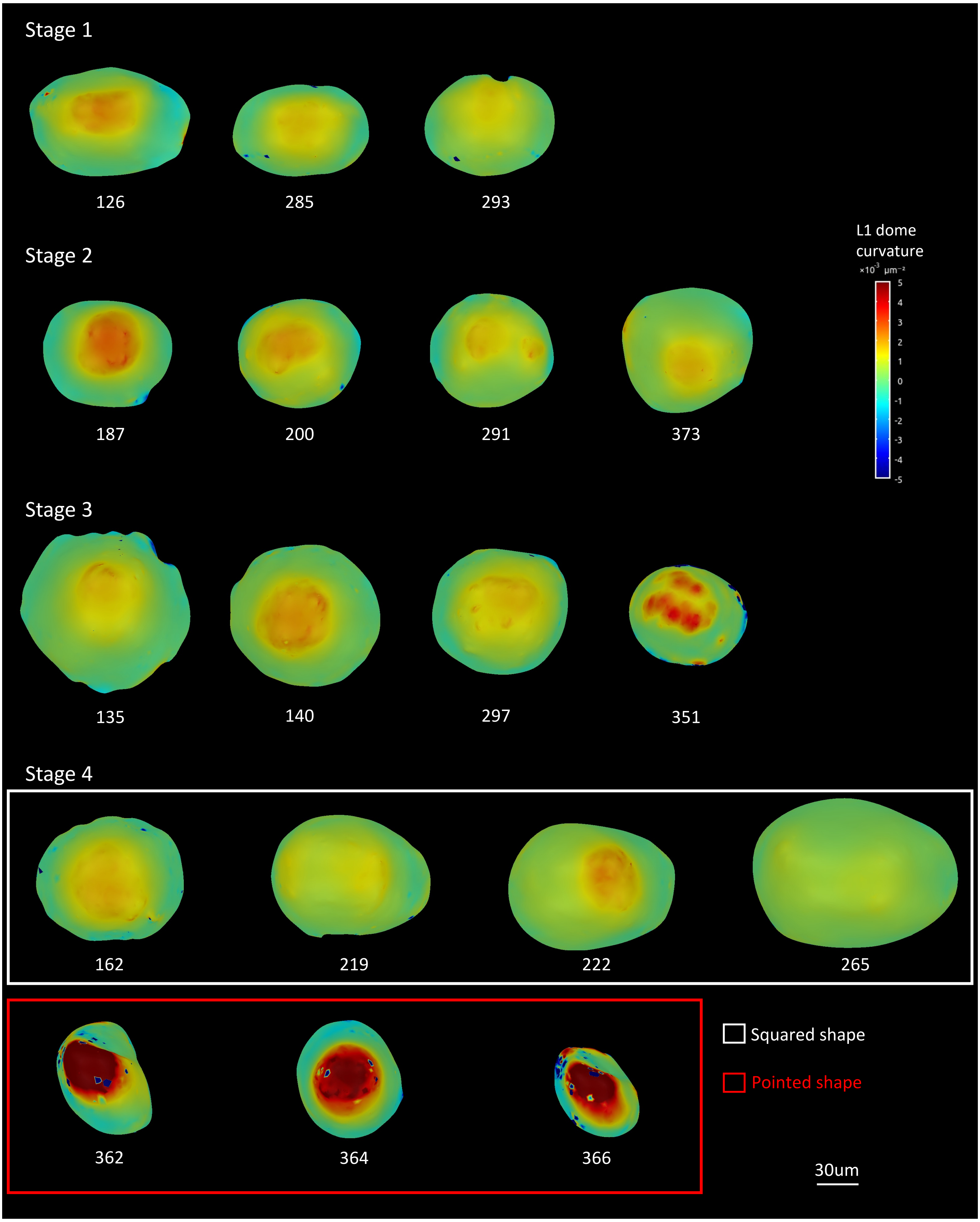

Supplementary figure 7. Gallery of ovule primordia curvatures.

For each developmental stage, with the exception of floret meristems, analysis of L1 dome curvature was performed on ovules surface meshes without irregularities. The curvature value is projected onto the mesh as a colored signal (blue: low curvature; red: high curvature). Numbers below images indicate organs' image stack ID. Scale bar: 30µm
