# Supplementary material for: 3D cellular morphometrics of ovule primordium development in Zea mays reveal differential division and growth dynamics specifying megaspore mother cell singleness
Source: Front Plant Sci. 2023 May 12;14:1174171. doi: 10.3389/fpls.2023.1174171 (PMC10213557; doi:10.3389/fpls.2023.1174171)
Supplement: Supplementary Figures — 1 to 7 (pdf file). [file DataSheet_1.zip › Supplementary Material/Ouedraogo_et_al_Supplementary_Dataset_1.pdf]

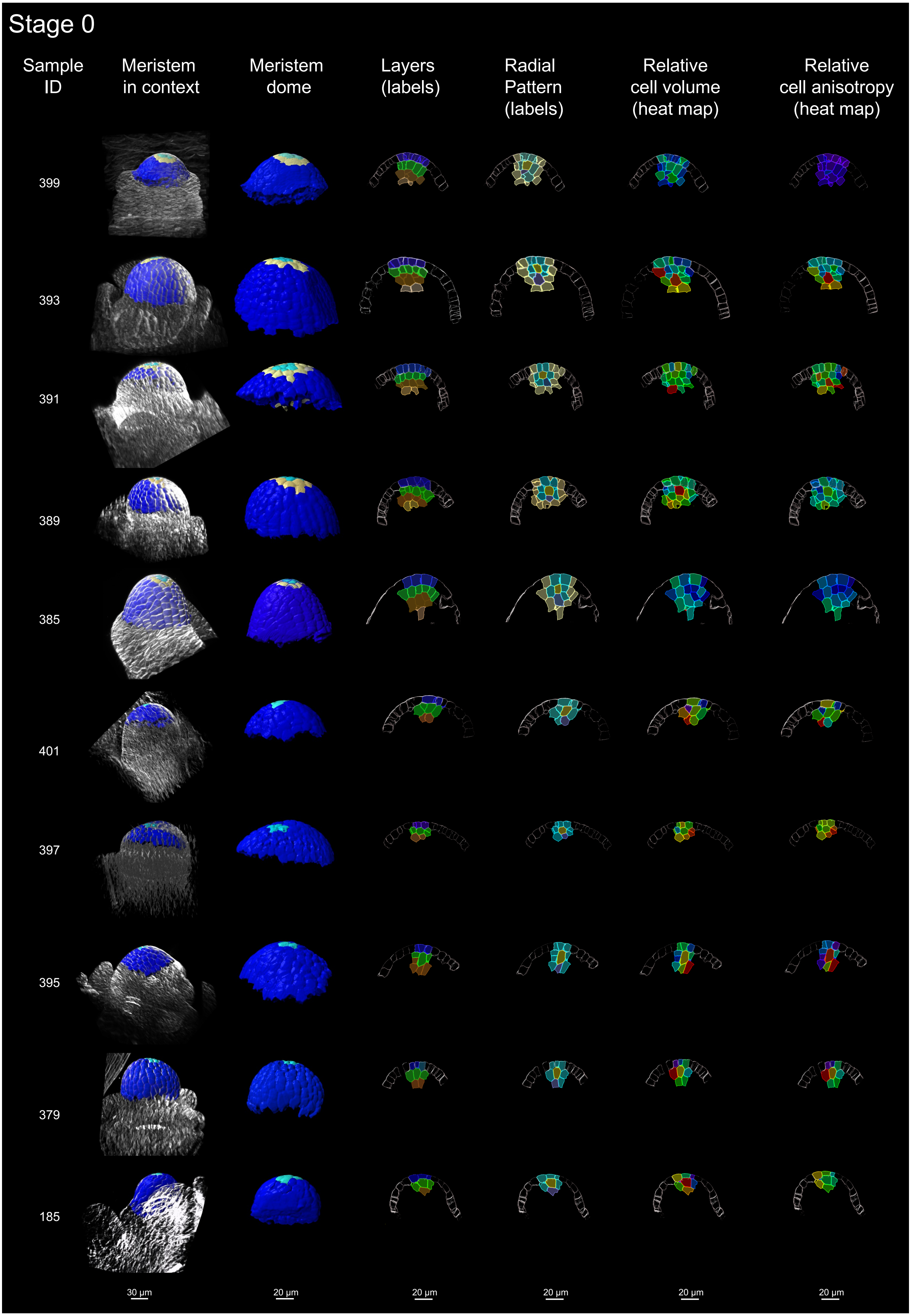

Supplementary dataset 1. Gallery of complete image collection of meristems (Stage 0) and ovule primordia (Stages 1 to 4). Organs are represented as described in Figure 1.

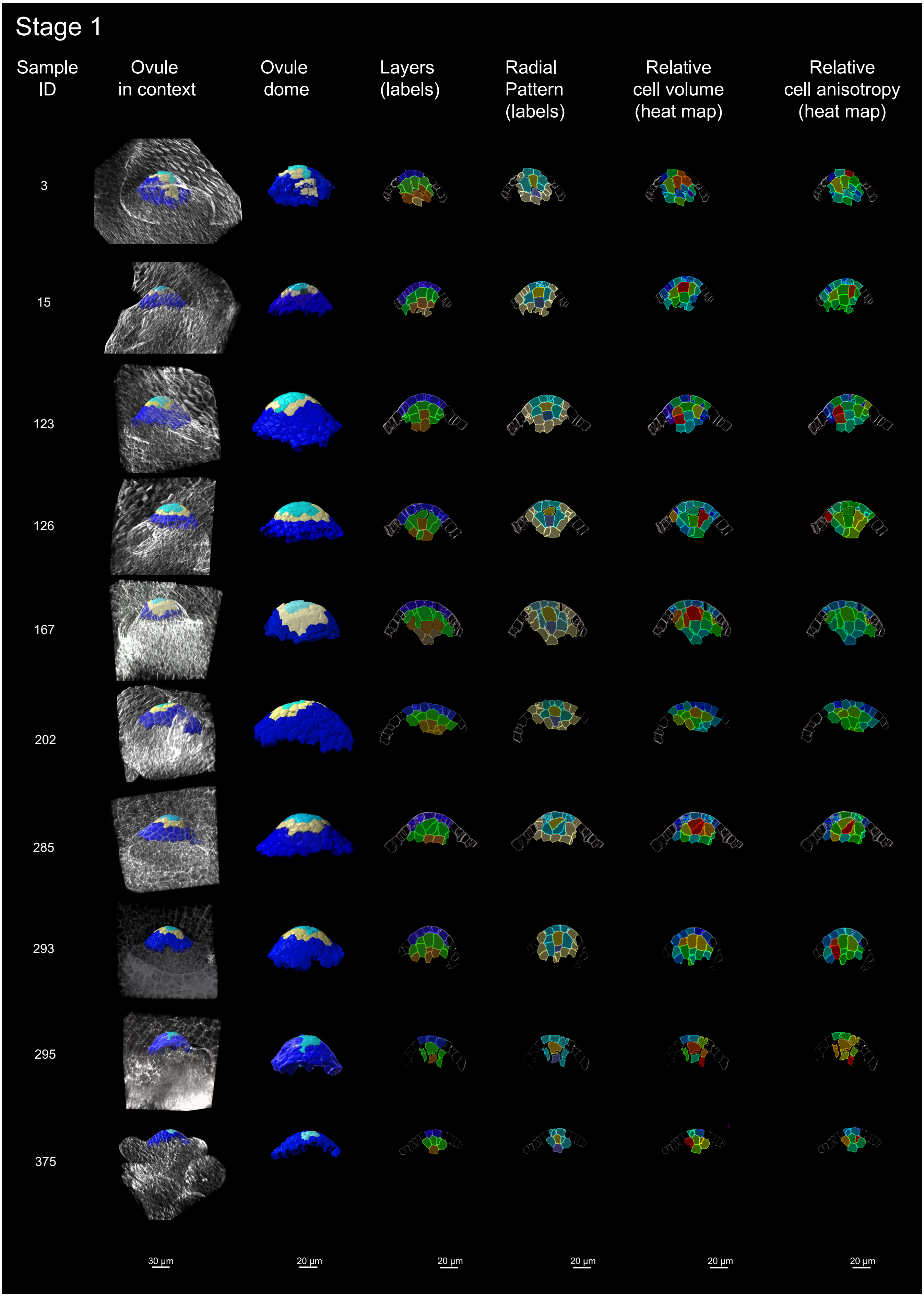

Supplementary dataset 1. (continued)

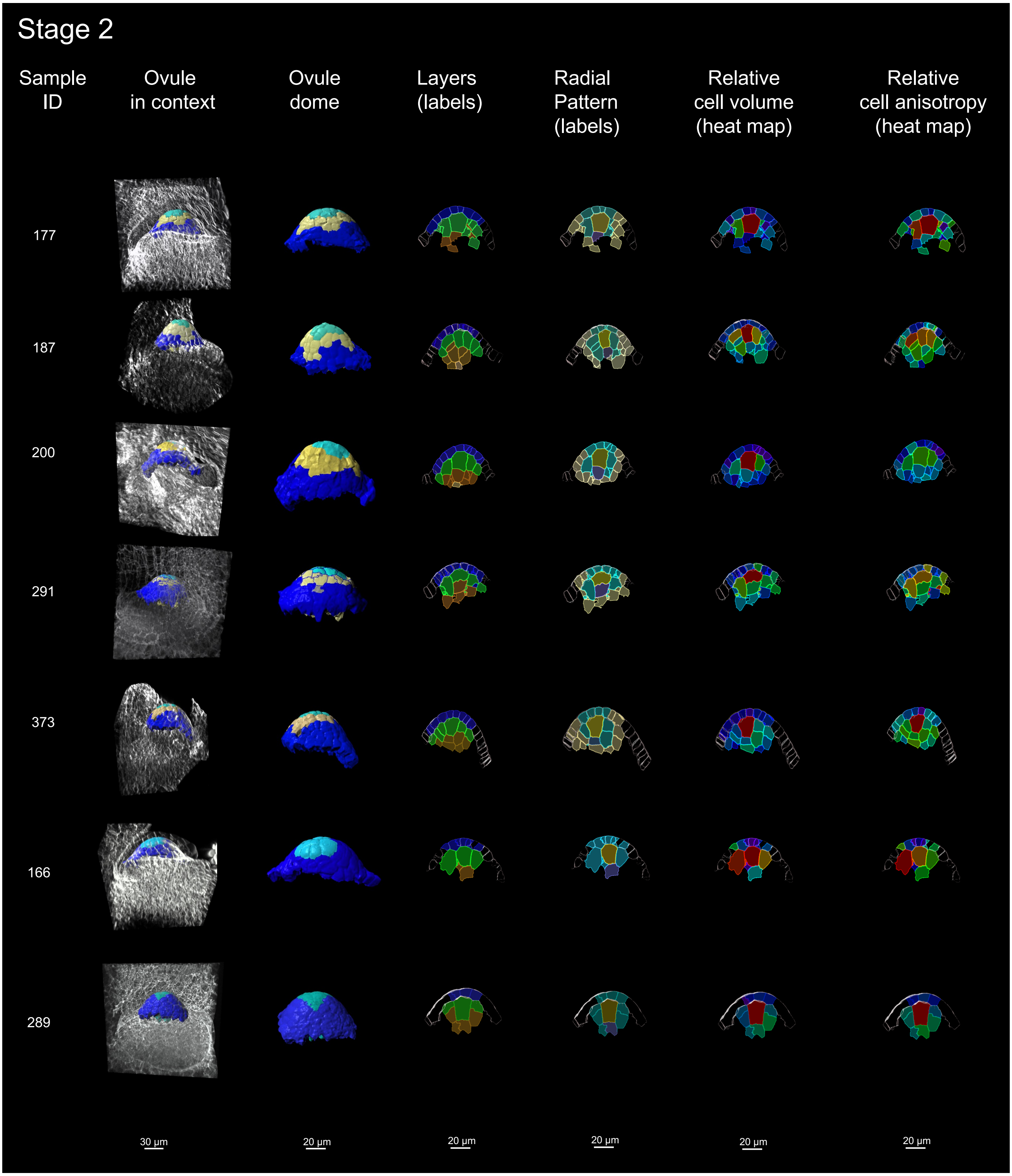

Supplementary dataset 1. (continued)

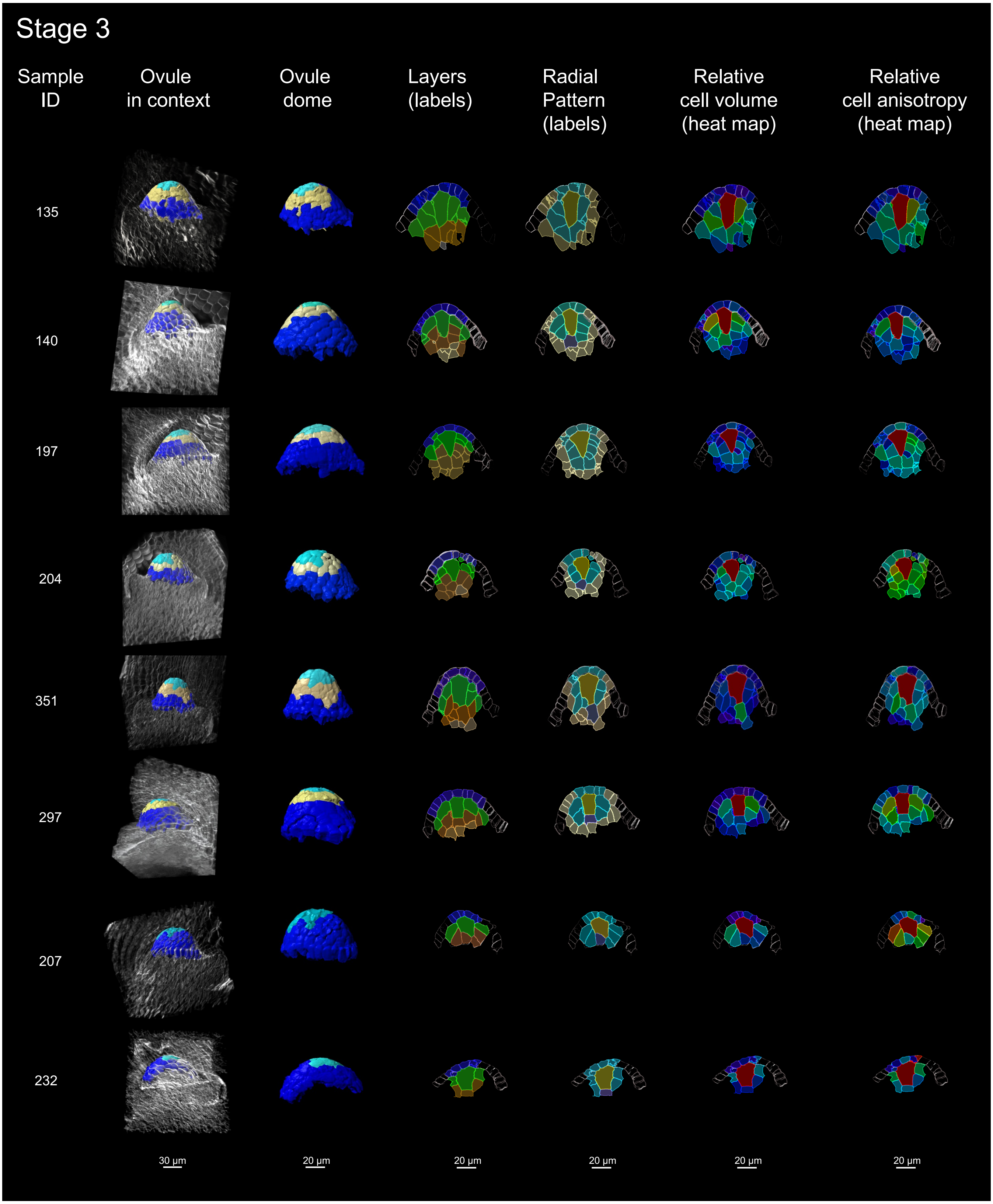

Supplementary dataset 1. (continued)

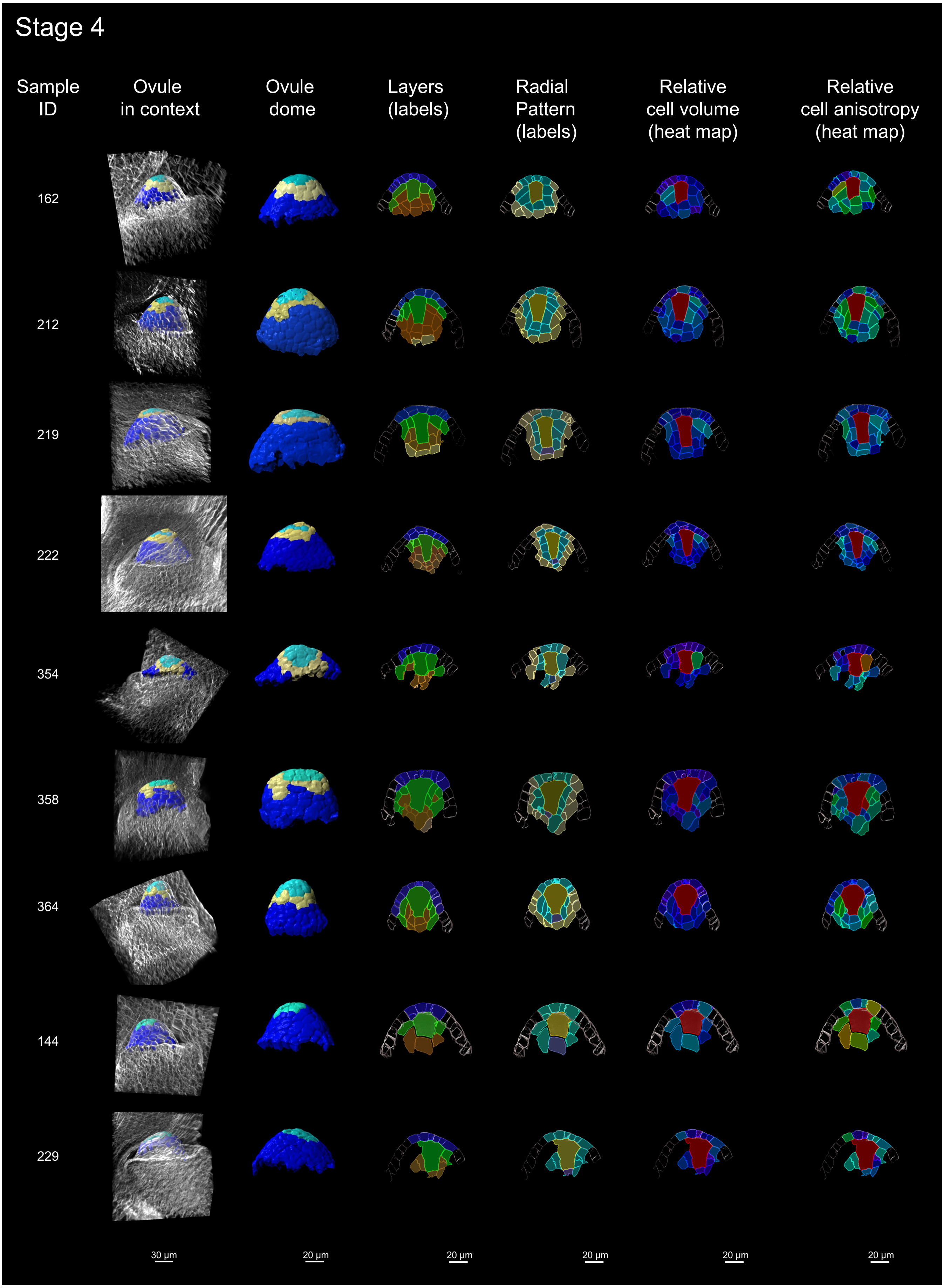

Supplementary dataset 1. (continued)

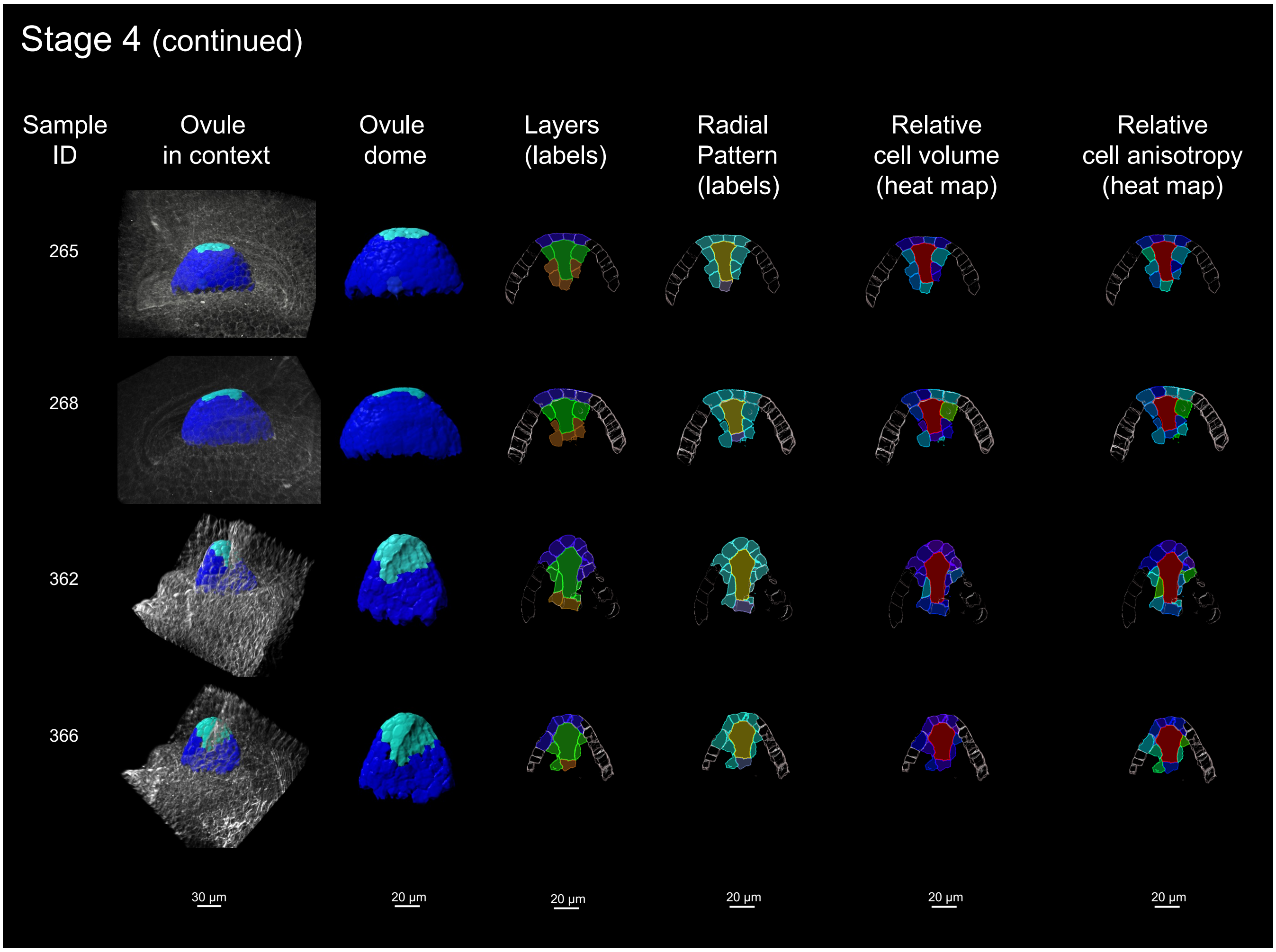

Supplementary dataset 1. (continued)
